# Development of a Smartphone-Based University Library Navigation and Information Service Employing Wi-Fi Location Fingerprinting

**DOI:** 10.3390/s21020432

**Published:** 2021-01-09

**Authors:** Guenther Retscher, Alexander Leb

**Affiliations:** Department of Geodesy and Geoinformation, TU Wien, 1040 Vienna, Austria; e1326712@student.tuwien.ac.at

**Keywords:** Wi-Fi positioning, navigation, location fingerprinting, RSSI-based positioning, probabilistic approach, information service, book tracking

## Abstract

A guidance and information service for a University library based on Wi-Fi signals using fingerprinting as chosen localization method is under development at TU Wien. After a thorough survey of suitable location technologies for the application it was decided to employ mainly Wi-Fi for localization. For that purpose, the availability, performance, and usability of Wi-Fi in selected areas of the library are analyzed in a first step. These tasks include the measurement of Wi-Fi received signal strengths (RSS) of the visible access points (APs) in different areas. The measurements were carried out in different modes, such as static, kinematic and in stop-and-go mode, with six different smartphones. A dependence on the positioning and tracking modes is seen in the tests. Kinematic measurements pose much greater challenges and depend significantly on the duration of a single Wi-Fi scan. For the smartphones, the scan durations differed in the range of 2.4 to 4.1 s resulting in different accuracies for kinematic positioning, as fewer measurements along the trajectories are available for a device with longer scan duration. The investigations indicated also that the achievable localization performance is only on the few meter level due to the small number of APs of the University own Wi-Fi network deployed in the library. A promising solution for performance improvement is the foreseen usage of low-cost Raspberry Pi units serving as Wi-Fi transmitter and receiver.

## 1. Introduction

In recent years, a number of technologies and methods have been developed and improved for indoor positioning. One of these technologies is based on the use of Wireless Fidelity (Wi-Fi). As such infrastructure is already installed in most public buildings and therefore costs are low, it is one of the most researched technologies for indoor positioning. Thereby positioning can be made either cell-based, as well as using lateration or fingerprinting. In particular, location fingerprinting has proven itself in practice. It is an approach to pattern recognition and based on received signal strength indicator (RSSI) measurements of the surrounding Wi-Fi Access Points (APs) in an off-line training and an on-line positioning phase. During the training phase, the RSSIs of the surrounding APs are measured in the area of interest at reference points to build-up a fingerprinting database, which can be visualized by signal strength radio maps. For the positioning in the on-line phase, the measured fingerprint is then compared at an unknown location with those in the empirically determined radio map. Finally, the position in the radio map that best matches the on-line RSSI measurement is returned. A major disadvantage of the empirical method, however, may be the time required to set up and maintain the database. In addition, the measurements must be carried out again during the installation of new transmitter or other structural changes. Another challenge is the large variation of the observed RSSI values due to signal fluctuations [1]. Despite these disadvantages, fingerprinting is nowadays one of the most popular method for an indoor positioning system (see e.g., [2,3]).

TU Wien (Vienna University of Technology) is the largest scientific-technical research and education institution in Austria. With its four inner-city locations as well as a science center further away from the city center, the University has more than 12,000 rooms in 30 buildings on an available area of approximately 269,000 m^2^. With such a large number of buildings and rooms, a positioning and navigation system can be a helpful tool. Especially in the large library building which has six levels with an area of 1160 m² the localization and tracking of the books is a challenging task. The motivation of this study is therefore to help students, employees, and visitors of the University to find at least the correct bookshelf. Furthermore, the individual books shall be located and tracked. For that purpose, also the use of Radio Frequency Identification (RFID) is foreseen. It was seen in the tests that not all areas in the library can be covered with Wi-Fi to guarantee the required localization accuracy. Thus, also the integration of Bluetooth is considered for the positioning and navigation system.

The paper is structured as follows: Section 2 provides a comprehensive survey of suitable indoor positioning techniques leading hereto to the chosen technical solution for the library navigation and information system. In Section 3 the specifications and characteristics of the test site and measurement procedures are introduced followed by a description of the analyses carried out for the off-line fingerprinting training phase in Section 4. Section 5 then deals with the impact on the results of different times required for the measurement of a single Wi-Fi scan, referred to as scan duration in the paper, in the kinematic measurement mode. In the following, Section 6 addresses the localization performance and achievable positioning accuracies in the on-line positioning phase. Section 7 pinpoints a useful strategy towards the development of a library navigation and information system. Finally, the paper is concluded and outlook on future work is given in Section 8.

## 2. Suitable Indoor Positioning Techniques Survey

At the beginning of the study at hand a survey was carried amongst solutions for indoor positioning to be able to select the overall best absolute positioning technology for the library navigation and information service. In this section first the requirements for an indoor positioning system (IPS) in general and in particular for the service at TU Wien are discussed. Suitable technologies are identified and assessed in a compendium where their characteristics and physical properties are analyzed in detail. Moreover, the selection process of the chosen technique is exemplified. An IPS should not only provide a certain required positioning accuracy, but also function reliably and be designed to be user-friendly. Moreover, attention should also be paid to data protection and costs.

### 2.1. General Aspects

An IPS is a wireless location system used to navigate, locate and position people and objects inside buildings. It usually consists of at least two hardware components, i.e., a transmitter and a receiver. One of the two components is always the mobile device to be located. Depending on the functionality of these two components, a first classification of positioning systems can be made into self- and remote-positioning [3]. In a self-positioning system, the receiver represents the mobile device that measures the signals of transmitters which coordinates are known. The position is then calculated on the mobile device. It is also possible that the measurement results are sent from the receiver to a master station. If the position at the master station is calculated, the positioning mode is referred to as indirect remote-positioning. In a remote-positioning system, the transmitter represents the mobile device and the receivers are fixed at known locations. Then the results of all measurements are collected in a master station and the position of the transmitter is calculated. If the measurement results are sent back from the receiver to the transmitter so that the position is determined on the mobile device, it is called indirect self-positioning.

### 2.2. Technological Requirements

Due to the complexity of the environment in a building, the limitation of direct line-of-sight (LoS), blocking of signals and multipath effects but also due to reliability, availability and cost of the required equipment, indoor positioning is a big challenge. Both in terms of accuracy, which ranges from the sub-meter level to several meters, and in terms of cost, the systems exhibit a wide range.

The required positioning accuracy of an IPS depends on the application. While positioning accuracy in the meter range is sufficient for many applications, it may be too inaccurate for a warehouse or a library, for example. The achievable accuracy depends, among other things, on the position determination method and technology used. The number of transmitters and receivers plays an essential role. Furthermore, attention should be paid to the dimension in which the position is to be determined. In a multi-story building, in addition to the location (horizontal accuracy), the floor (vertical accuracy) must also be determined. For a library such type of accuracy is needed so that the user is able to find a certain bookshelf. With a bookshelf distance of 2 to 3 m the accuracy should be at least in this range. Since the building has several floors, it should be possible to determine the floor in addition to the location.

In terms of reliability and coverage of the positioning system it should always be available and consist of stable components so that localization can be performed in real-time. In addition, smaller signal failures should be compensated and a seamless transition between outdoor and indoor areas should be possible. This requires a sufficient number and a good distribution of transmitters or receivers. Due to the widely distributed locations of the TU Wien, the positioning system should work on the entire inner-city campus, both outside and inside buildings. The aim is to identify the building in which the user is located or whether he/she is outside the campus.

Any student, staff member or visitor of the University should be able to operate the positioning system easily. With the use of a smartphone being ubiquitous these days, an application for these devices is a useful solution for the navigation and information service. The presentation of information should be adapted to the user and consideration should also be given to physically impaired persons. In addition, care should be taken to ensure that the application consumes as little energy as possible. The power consumption depends, among other things, on whether the position is calculated on the smartphone or externally. If the position is determined on an external server, it can usually be calculated much faster. Positioning should also not take too long, but should be performed in real-time if possible. This means that the latency—the time it takes for the user to see their location—must be also as short as possible.

Privacy is a major challenge for an IPS, as not all people want to share their current location. Therefore, it is also important to consider the privacy and security of IPS users. Therefore, the IPS operator should ask how the user can trust the system [4]. The decisive factor here is where the position is determined, i.e., either on the mobile device or in a master station or on an external server. If the location is determined directly at the mobile device, no forwarding of information to the IPS operator is required, thus ensuring the privacy of the mobile user. However, determining the position on the mobile device again consumes a lot of energy and requires sufficient computing power, so it is significant to reduce the computational complexity of the IPS [5]. In [6], different IPS are compared and it was found that most of the commonly employed technologies present data protection problems. Only remote positioning technologies, such as the use of inertial sensors, provide a high level of data protection. Their disadvantages, however, are their low precision due to accumulation of errors leading to high sensor drift rates. Furthermore, they require an absolute positioning method in addition for determining the start location and an update of the only relatively determined positions.

Last but not least, the cost of an IPS is one of the basic decision criteria and depends on several factors, such as available money, time, infrastructure and energy. The time factor is related to installation and maintenance. Also the costs for software, server and maintenance of databases have to be considered. The number of transmitters or receivers are thereby considered as infrastructure costs. However, it is not always obvious what the actual cost of an IPS will be. For example, it can be assumed that a Wi-Fi-based IPS does not incur hardware costs because the required Wi-Fi APs are already available. This is valid, however, for RSSI-based solutions at TU Wien only. New approaches based on measurement of the ranges to the Wi-Fi APs require the installation of new hardware. 

Power consumption is also a decisive cost factor of a positioning system. Some devices are completely energy passive, such as passive RFID tags. These devices only respond to external fields and can therefore have an unlimited lifetime. Other mobile devices, on the other hand, only have a few hours of battery life without recharging [3]. For TU Wien the positioning system should not cause any costs for the user, so he/she should not have to buy new hardware. For indoor applications, technologies should be used that are not too expensive to purchase and install. For outdoor use, GNSS positioning can be used if enough satellite signals are available. 

### 2.3. Compendium of Common Technologies

Different signals and technologies can be used for positioning of a mobile device. In order not to go beyond the scope of this work these technologies are presented briefly in the following, with a closer look at their characteristics and physical properties as well as their advantages and disadvantages. A first division can be made into optical, sound-based and radiofrequency technologies, magnetic fields and inertial sensors.

Optical technologies work with signals that are in the visible or infrared range. This corresponds to electromagnetic radiation with wavelengths from 380 nm to 10 µm. Unlike radio signals, infrared signals and visible light cannot penetrate walls and other obstacles, limiting positioning to enclosed spaces. Transmitter identification can be modulated on the infrared signal, which allows different transmitters to be distinguished. Therefore, the position determination can be carried out with all common measuring principles. One of the first IPS to use infrared signals is Active Badge [7]. Technologies that use visible light for data transmission are also called Visible Light Communication (VLC) [8,9]. For instance, light-emitting diodes (LEDs) can be used. The transmission of data using LED is possible because the light source can be switched on and off again in very short intervals. This flickering can be so fast that it cannot be perceived by human eyes. A variety of modulation methods can be used. The principle for VLC is that each fixed LED lamp has a different flicker coding, so that the mobile sensor (e.g., the smartphone camera) receives the light and compares the modulation with the known coding scheme [10].

The position of a user can also be determined by using acoustic signals. The time of the broadcast can be determined by simultaneously sending a radio and an acoustic signal. Since the radio signal arrives earlier than the acoustic signal at the receiver, the difference between these two times can be used to calculate the range. For this process, however, an exact synchronization of the transmitter and receiver clocks is necessary [11]. Due to the fact that acoustic signals travel with slower speed than infrared or radio frequency signals the acoustic signals travel time measurement allows for higher accuracy. The propagation velocity thereby depends on the energy of the signal as well as on the density and temperature of the medium it penetrates. The sound-based technologies can be divided into audible [12] and non-audible sound (ultrasonic signals) [13]. The position can be determined by all range-based measurement principles (see Section 2.4). Nakashima et al. [14] determine the position using a digital watermark that they inserted into the audio track of each speaker. However, this technology is difficult to implement in reality, as it can be assumed that there is a lot of noise in a building, making it difficult to determine the position. In addition, audible noise—especially in Universities libraries—can be very annoying in everyday life. Thus, ultra-sonic signals are usually employed for sound-based positioning. With this technology, the travel time of emitted ultrasonic pulses is usually measured and the position subsequently determined by means of lateration. For example, in [13] the position is determined using Time of Arrival (ToA), while in [15] localization is performed using Time Difference of Arrival (TDoA). The signals can be either transmitted by the mobile device and received by permanently installed receivers or vice versa. The mobile device can also be a transmitter and receiver at the same time, so that the distance can be determined by Round Trip Time (RTT) measurements, which means that no additional infrastructure is required. In addition to the multipath effect, another problem with this technology is that ultrasonic systems are very sensitive, since even a low noise already generates ultrasonic waves and thus interferes with the system. Among the commonly known systems are the Active Bat [16], Dolphin [17] and Cricket [18]. Lopes et al. [15] proposed a reliable acoustic indoor positioning system fully compatible with a conventional smartphone. Thereby acoustic ranging in the audio band based using non-invasive signals is carried out using the smartphone audio I/O. In order to support the positioning system a Wireless Sensor Network (WSN) of synchronized acoustic beacons is used for TDoA ranging. They achieved an absolute positioning error of less than 10 cm on the 95% significant level in their tests.

Unlike infrared signals and visible light, radio signals can penetrate walls and other obstacles, making positioning not limited to enclosed spaces. Each station that transmits radio frequency (RF) signals has a unique address by which it can be identified. The most popular RF technologies are Wi-Fi, Bluetooth, Radio Frequency Identification (RFID) and Ultra-wide Band (UWB).

A Wireless Local Area Network (WLAN) transmits electromagnetic signals over the free 2.4 and 5 GHz Industrial, Scientific and Medical (ISM) band with wavelengths of 12.5 and 6.0 cm, respectively. WLAN or Wi-Fi is based on the IEEE 802.11 standard. Indoors the signals have a range of 20 to 100 m [19] and can also pass through walls. Positioning can be performed using time-based methods (ToA and RTT), Angle of Arrival (AoA) or RSSI. The specifics of Wi-Fi are further discussed in Section 2.6.

Bluetooth is also an electromagnetic signal, which has a wavelength of approximately 12.5 cm in the frequency range between 2.402 and 2.480 GHz. The latest Bluetooth version (5.1) now has a range of approximately 200 m. In addition, from this version onwards, the direction angle of the received or transmitted signal can also be measured using AoA [20]. Position determination via Bluetooth can also be time-based or RSSI-based. However, since Bluetooth transmits in the same spectrum as Wi-Fi, it is susceptible to interference. Advantages of Bluetooth are availability as it is supported by most smartphones, low cost, and low power consumption, which allows transmitters to run on battery for several months or even years [21]. It has been considered by the IT Department of TU Wien to use Bluetooth Low Energy (BLE) Beacons in addition on the campus for areas with limited or no Wi-Fi coverage. However, one must also take into account the signal attenuation, the multipath effect and the fluctuations in signal strength while using Bluetooth. Zhao et al. [22] demonstrate that BLE is more accurate than Wi-Fi for localization when lateration approaches are employed where RSSI values have to be converted into ranges.

Typical RFID frequency ranges are 125–134 kHz for low frequency, 13.56 MHz for high frequency and 860–890 MHz for ultra-high frequency (UHF) [23]. In RFID, a reader communicates with one or more transponders (so-called tags) using radio waves. If a tag comes near a reader, the two start communication and can exchange information with each other, such as the location of the permanently mounted component. The tags or readers are then mounted at strategically important locations inside the building (e.g., at entrances). Communication takes place either by inductive coupling or electromagnetic waves [11]. RFID tags can be classified according to whether they are passive, semi-passive or active [24]. Passive tags do not have their own power supply and respond with the energy the reader releases with the help of a small antenna. They are much lighter, smaller and cheaper than active tags, so they only have a range of 1 to 2 m. A passive tag can be attached to a mobile device or a book, for example, which is read by permanently installed readers. Thus, they can be used for cheap book labelling, e.g., also to replace barcodes. In the TU Wien library it is thought to use this technology. RFID can then also serve as a security function by installing readers at the building exits, which give an alarm signal if a book leaves the library unauthorized.

UWB is based on the transmission of electromagnetic waves by a sequence of very short pules (less than 1 ns) with a wide bandwidth of larger than 500 MHz. This allows reflected signals to be better filtered, minimizing the multipath effect and improving positioning accuracy, which is one of the major advantages of this method [25]. Unlike other RF-based technologies, UWB devices can also transmit signals in multiple frequency bands simultaneously. Another advantage is the lower power consumption. However, UWB devices are still more expensive to purchase and install. Position determination can be performed using lateration (ToA and TDoA) and/or angulation (AoA) [11,19]. 

Other RF-based localization technologies include Zigbee and Long-range Wide-area Network (LoRaWAN). Zigbee is a common low power technology, often used in Internet of Things (IoT) applications with same ranges and coverage as the aforementioned technologies. LoRaWAN, on the other hand, can reach ranges of up to 15,000 m transmitting at 915 MHz, which may allow a significant reduction of transmitters in order to cover an environment. In the tests conducted by Sadowski and Spachos [26] it was seen, however, that this technology showed the worst performance for indoor localization. Zigbee has a similar low energy requirement to LoRaWAN, while its performance is much higher. The authors further mention that BLE is a low power and cost efficient solution for IoT localization in small crowded areas. Wi-Fi, however, consumes the most power out of all the examined technologies, but is advantageous because of its high ubiquity. It also achieved the highest positioning accuracies in their tests. These results confirm that a localization service based on Wi-Fi is the way to go ahead for our application.

An indoor position determination can also be performed by measuring the magnetic field strengths. Both the geomagnetic field [27] and an artificially generated magnetic field [28] can be used. Although there are some approaches using the later, most modern systems use the Earth’s magnetic field strength [11]. Using the embedded magnetometer in a smartphone magnetic field fluctuations can be measured as the magnetic field shows local anomalies, which are caused by objects, such as electrical devices and cables and building structures, such as concrete reinforcement. Assuming that these anomalies within a building are nearly static and have sufficient variability, they provide a unique magnetic fingerprint such that localization can be carried out with fingerprinting (see Section 2.5) [29]. A drawback, however, is that magnetic fields are already disturbed by small changes in the environment, e.g., caused by people, which complicates localization. 

Global Navigation Satellite Systems (GNSS) chipsets are an integral part of every smartphone. At least the US Navstar Global Positioning system (short GPS) is supported in smartphones. In addition, more and more smartphones include the measurements from the Russian GLONASS, the European Galileo and the Chinese Beidou. The GNSS signals are in the L-band, i.e., in the frequency range from 1.164 to 1.300 MHz with a wavelength from 25.7 to 23.1 cm and 1.559 to 1.610 MHz with 19.2 to 18.6 cm wavelength. In this work GNSS, is used only for outdoor positioning, but it is out of the scope of this paper to further discuss its application in the TU Wien localization service.

The users’ location can also be determined relatively from a given start position with the help of inertial sensors, which are embedded in every smartphone. They include Micro-Electro-Mechanical Systems (MEMS)-based accelerometers and gyroscopes. The measurements of the accelerometer can be used to obtain the distance travelled, such as from step counting, and the gyroscope is used to estimate the direction of movement of the user. The employed location technology is dead reckoning and in case of pedestrian navigation it is referred to as Pedestrian Dead Reckoning (PDR). Due to the large error drift of MEMS-based sensors, which accumulates over time, a combination with absolute positioning techniques is required to update the measurements (see e.g., [30,31]). Using filtering, such as with a Kalman or particle filter, is another popular approach to reduce sensor drift rates and estimate the current users’ location (see e.g., [32]). In this work, the use of inertial sensors is not foreseen at this stage as the sole use of an absolute positioning technology is evaluated.

Additionally, it is worth mentioning that more and more smartphones also have a pressure sensor built in, which can be used to measure the air pressure and thus determine the altitude. This can be particularly useful in multi-storey buildings as the sensor can be used to determine the current floor (see e.g., [33,34]).

Smartphone cameras provide visual information in addition to the position, but computational-intensive image recognition software is required for localization. One approach for smartphone positioning is scene analysis. Similar to the fingerprinting method, a database is first filled with images of the environment and linked to the respective location. After that, a photo can be taken from the smartphone user’s point of view and this is then compared with the images from the database to determine the position [35]. The big advantage thereby is that no signals are used for position determination and therefore the effects of signal propagation do not play a role. Moreover, no additional infrastructure needs to be installed. A disadvantage, however, is the large amount of time required to set up and maintain the database, which has to be updated when structural changes occur. If the position is not determined on the mobile device, the image must first be transferred to the master station, which means a large data transfer. On the other hand, if the position is to be computed on the mobile device, it requires a large amount of RAM since the image recognition software packages are computationally intensive. However, due to the recent technical developments with improved image recognition algorithms and computational capabilities as well as greater data transmission rates, these drawbacks have been minimized [36]. Another method is called visual odometry [37,38] where the self-motion (translation and orientation) of a person or object is determined using single or multiple cameras attached to the mobile object. Thereby the images must contain sufficient meaningful information, such as color, texture, shape, etc., to estimate the movement of the camera. Visual odometry offers a good trade-off between cost, reliability, and implementation complexity [39] and is widely used in mobile robotics [40].

Table 1 provides a comparison of the most commonly employed technologies in terms of their advantages and disadvantages as well as respective costs and Table 2 summarizes the characteristics of different suitable positioning methods. As with these technologies, however, it is somewhat difficult to give an exact positioning accuracy figure, as it depends heavily on the measurement principle, method and infrastructure used, and is therefore not mentioned. Table 3 provides rough ranges of achievable positioning accuracies for the different useable positioning methods. The cost of each technology here depends mainly on the infrastructure already in place. From the user’s point of view, it is assumed that a smartphone is used for positioning and therefore no costs are incurred. The costs in Table 1 therefore relate only on the installation and maintenance of the infrastructure in the building. Since an IPS is used in various applications, there is no one method or technology that is superior to the others, but only one that best meets the requirements set, both technically and economically. As each of the techniques presented has different advantages and disadvantages, a combined solution is the best way to overcome the drawbacks of each individual method and reduce measurement errors. If several positioning technologies are combined, the system is also referred to as hybrid IPS. 

From Table 2 the following conclusions can be drawn: Cell-of-Origin (CoO) based positioning is only suitable for determining a first approximate solution. Unlike location fingerprinting and scene analysis, lateration and angulation do not require an off-line training phase. With these two methods, however, care must be taken that the receiver measures the LoS signal and not a reflected signal. Angulation also requires an antenna array or a directional antenna to measure the incident angles. The major advantage of fingerprinting is that it is more resistant to multipath than lateration and angulation. In addition, there is no need LoS between the transmitter and receiver. The major disadvantage, however, is the time required to set up and maintain the training fingerprint database. The advantages and disadvantages of scene analysis are similar to those of fingerprinting. An additional disadvantage is the large amount of data that has to be transmitted if the position is to be calculated on an external server. If the position is determined on the smartphone, however, sufficient computing power is required, since the image recognition software packages are computationally intensive. Dead Reckoning (DR) is the only method based on relative positioning included in the Table. The big advantage over the other methods is that it only requires a smartphone whit its embedded inertial sensors. As already aforementioned, smartphone sensors show high drift rates leading to low positioning accuracies which are steadily increasing in time. 

### 2.4. Range-Based Localization Operational Principle

The most common measuring principles for position determination using ranges between a transmitter and a receiver are briefly reviewed in the following. Mostly it is assumed, however, that only the horizontal position coordinates must be determined. As aforementioned it is also important to locate the user on the correct floor in a multi-storey building. The techniques have in common that the mobile device can be either the transmitter or the receiver. These measurement principles form the basis for the methods of localization.

To derive the ranges between a transmitter and receiver several methods are applicable, such as Time of Arrival (ToA) (also referred to as Time of Flight (ToF)), Time Difference of Arrival (TDoA) and Round Trip Time (RTT) measurements [36]. In addition, ranges may be derived from RSSI measurements using so-called path loss models (see e.g., [41] for examples of common path loss models). These models are describing the relationship between RSSI values and distance by assuming that the RSSI decreases with increasing range from the transmitter.

For lateration, the individual ranges between the transmitters and the mobile device are first estimated. The methods ToA, RTT, TDoA and RSSI can be used for this purpose. At least three ranges must be measured for unambiguous localization in 2D. This is referred to as trilateration. If more ranges are used it is called multi-lateration. For the position determination the intersection is calculated from the distance radii. The location of the transmitter is in the center of a circle in 2D and a sphere in 3D (see e.g., [1]).

Table 3 summarizes the main properties of range-based localization techniques. The main disadvantage of the time-based methods (ToA, RTT and TDoA) are that an accurate time synchronization is required and such an error would have a large impact on the position determination. In addition, care must be taken not to measure the reflected signals, but rather the LoS signals, in order to obtain accurate results. The largest disadvantage of the RSSI-based lateration method is that the measured signal strengths can vary by a large extent. However, there is no need for a LoS between transmitter and receiver and that the multipath does not play a major role. Furthermore, no time synchronization between the two components is necessary. The levels of achievable positioning accuracies provided in the Table are also representative for the use of Wi-Fi in RSSI- and RTT-based lateration solutions.

### 2.5. Location Fingerprinting

Fingerprinting is a pattern recognition approach based on the RSSI measurement principle. The method consists of the training phase (or off-line phase) and the positioning phase (on-line phase). During the training phase the RSSIs of the surrounding transmitters are measured at several reference points in space and saved in a multi-dimensional database which can visualized in radio maps. The radio maps can be stacked into so-called datacubes as proposed by Retscher [1]. The radio map datacubes are the 3D arrays of the radio maps of the sensed APs at a certain location. The datacube has two spatial axes and a vertical AP axis. It is created by stacking radio maps vertically onto each other allowing the examination of the interrelations of the three quantities easily. For positioning in the on-line phase, the measured fingerprint at an unknown location is then compared with those in the empirically determined radio map datacubes. Finally, the position in the radio map that best matches the on-line measurement is returned. The radio map can also be created using simulated models taking into account the signal propagation in the area of interest; but this can be very complex.

In contrast to lateration, fingerprinting uses the signal attenuation and the multipath effect to determine the position. In addition, there is no need for a direct LoS between transmitter and receiver. One disadvantage of this method is the time required to set up and maintain the fingerprint database. In addition, training measurements must be carried out again when a new transmitter is installed or when structural changes are made [42]. Another challenge is the large variation in the observed RSSI values due to signal fluctuations. Despite these drawbacks, fingerprinting is now one of the most popular methods for an IPS. The specifics regarding the use of Wi-Fi fingerprinting are reviewed in the following section.

### 2.6. Specifics of Wi-Fi Positioning

Smartphone-based positioning using Wi-Fi plays a dominant role in the indoor positioning field and thus, it has become increasingly popular. This section presents briefly the Wi-Fi specifications and the properties of the two most commonly employed techniques in Wi-Fi positioning, i.e., the location fingerprinting and lateration-based approaches.

A Wi-Fi AP broadcasts small packets, i.e., the beacons, containing the Service Set Identifier (SSID) and the Media Access Control (MAC) address approximately every 100 ms. This ensures continuous data transmission. An AP can also transmit several signals simultaneously, with each signal belonging to its own Wi-Fi network. Furthermore, it is important that different channels are assigned to the APs, otherwise interference will occur [43]. The mobile device receives the signal and can identify the AP by the MAC address. The RSSI can additionally be sensed with various smartphone applications. Since the RSSI, the SSID and the corresponding MAC addresses can be accessed without any authenticated connection, this information is freely available. This allows wireless positioning to be performed autonomously, avoiding also privacy concerns that typically arise with other positioning technologies [31]. The size of the covered radio cell depends on the transmission power and the spatial conditions of the environment. Here, fluctuating influences such as the humidity in the air and in the building structure play a major role.

Wi-Fi is based on the IEEE 802.11 standard, which was developed by the Institute of Electrical and Electronics Engineers (IEEE). Since its introduction, several extensions have been developed, each with its own characteristics, such as the frequency band used or the range. Two frequency bands, i.e., the 2.4 and 5 GHz band, are available for Wi-Fi. The frequency range in the 2.4 GHz band (2400–2483.5 MHz) is divided into 14 channels, with only the first 13 channels used in Austria. Although the channel spacing is 5 MHz, a radio connection requires a bandwidth of 20 MHz (or at 802.11b 22 MHz). In order to avoid interference, therefore, in the case of spatially overlapping cells, overlapping-free frequency ranges with a distance of four channel numbers must be selected. The legally regulated maximum transmission power for the 2.4 GHz band in Austria is 100 mW. A total of 19 channels are freely available in the 5 GHz band. The frequency range 5150–5350 MHz may only be used with a maximum transmission power of 200 mW. The lower frequency range of 5150–5250 MHz may also be used with automatic power control, i.e., Transmit Power Control (TPC). TPC reduces the transmission power depending on the need. For example, if there is good connection between the devices, the transmission power is reduced. The 5470–5725 MHz frequency range may only be used outside buildings using TPC and Dynamic Frequency Selection (DFS) and with a maximum transmission power of 1000 mW. With the help of DFS, the AP automatically detects other radio systems and can switch to another frequency. This ensures that radar installations, satellite positioning services are not interfered [44]. The combination of TPC and DFS thus allows APs to determine the channels with the best availability and to use the lowest possible transmission power. The user therefore only receives the transmission power required for the current distance to the AP.

Each frequency band has its own advantages and disadvantages. In principle, the higher the frequency, the shorter the range (due to the higher signal attenuation). The 2.4 GHz band thus theoretically has a larger range, as it overcomes shielding materials with a lower loss. However, it has the disadvantage that the frequency band is compatible with other electronic devices or needs to be shared with other radio techniques, such as Bluetooth, microwave ovens, radio remote controls, etc., making it more prone to interference. The advantage of the 5 GHz band is the significantly higher data transmission rate, which does not matter with an IPS, since no data is transmitted as only the RSSI are measured. The big disadvantage of the 5 GHz band is that the signal is more shielded by walls. In the conducted tests it is seen that the 5 GHz Wi-Fi signals have a shorter range than the 2.4 GHz signals due to these properties (see Section 4.3). The Wi-Fi antennae in the APs bundle the electromagnetic waves and can thus influence the signal. Depending on the design of the antenna, the range and direction of the signals can be controlled and thus also the size of the radio cell. Commercially available APs have usually a range of 20 to 100 m within a building.

Due to signal damping and attenuation as well as signal fluctuations and noise, Wi-Fi positioning is normally not robust against dynamic changes in the environment. Thus, location fingerprinting is most commonly employed localization technology. For fingerprinting deterministic and probabilistic approaches can be employed. On the other hand, for lateration methods based on the measurement of the RSSI and RTT can be used. In the case of RSSI-based lateration, however, lower positioning accuracies are achievable in comparison to the RTT measurements with the new Wi-Fi IEEE 802.11mc standard. In Retscher [1] a comprehensive review of these techniques and the common mathematical models may be found. In this study, the available Wi-Fi AP hardware in the library of TU Wien supports only RSSI-based solutions. Thus, the chosen localization technique in this work is fingerprinting. A probabilistic fingerprinting approach is employed as they usually provide higher positioning accuracies than deterministic methods (see Section 6.1).

## 3. Test Site and Measurement Procedures

### 3.1. Test Site

**Figure 1:**
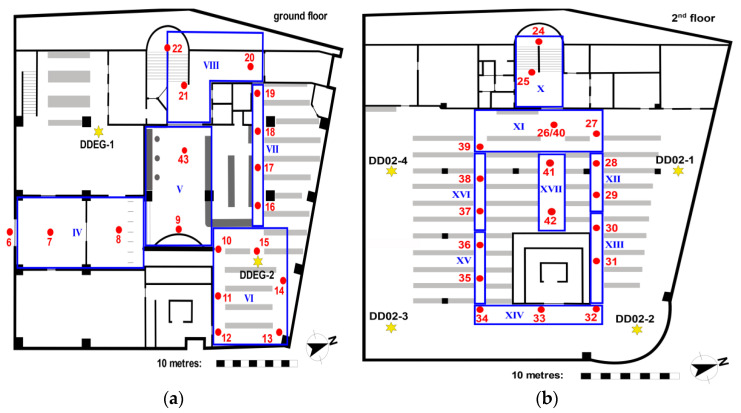
Ground floor (**a**) and reading room (**b**) of the library on the second floor showing the location of the APs (yellow stars), the checkpoints (red points) and the cells (blue Roman numbers). The bookshelves are represented as grey lines.

The University library of TU Wien is a multi-storey building and is connected with another large office building referred to as ‘campus Freihaus’. The chosen trajectory in the library with its waypoints on the ground and second floor is shown in Figure 1. It starts from outdoors in front of the main entrance (waypoints 1 to 5, not shown in the Figure) and has a length of around 379 m. Partly on the ground floor (points 10 to 19) and on the second floor (points 27 to 42) the trajectory runs along bookshelves. The reading room on the second floor is an open space of a size of approximately 830 m^2^. The layout of the bookshelves is illustrated with grey lines in Figure 1. The trajectory waypoints, also referred to as checkpoints, were placed at important passages and at the bookshelves on every second row. The distances between the checkpoints are therefore approximately 3 to 8 m. The number of visible APs was quite low as on the ground floor only two APs and on the second floor only four APs of the University Wi-Fi network are located (see yellow stars in Figure 1 for their location). Throughout the whole library, only four APs which are almost at the same location in the reading rooms as on the second floor can be found on each of the five floors. That is why, the second floor was chosen as major study area as this floor is representative for the whole library building apart from the ground floor. The low number of APs results in challenging conditions for matching of the fingerprints in the on-line positioning phase due to the small number of AP in close proximity.

Measurements were carried out during normal opening hours of the library with many people around. The users walked along the trajectories with an average walking speed of 1 ms^−1^ in both ways back and forth taking around four minutes each for the whole trajectory. Apart from measurements in kinematic mode, also stop-and-go and static observations were carried out along the trajectory and on the checkpoints. For the analyses, also cells (denoted with blue Roman numbers in Figure 1) were defined consisting of different numbers of checkpoints in dependence of the local spatial conditions. If several checkpoints are part of these cells a higher localization performance can be achieved as demonstrated in Section 6.2.

### 3.2. Wi-Fi Signal Availabilities

**Figure 2:**
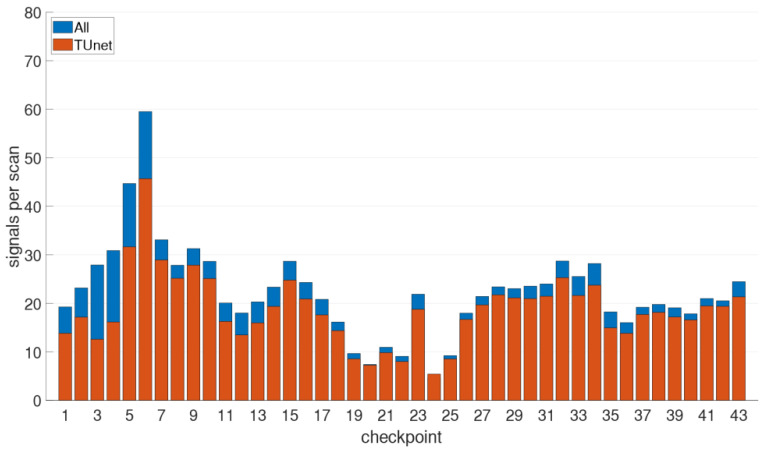
Average number of visible signals per scan on all checkpoints of the trajectory leading from outside through the ground floor to the reading room on the second floor.

Apart from the Wi-Fi University network in total six APs on the ground floor and 41 APs on the entire second floor providing signals could be sensed. Figure 2 shows the average number of visible Wi-Fi signals per scan on all checkpoints of the trajectory leading from outside through the ground floor to the reading room on the second floor. Note, that these numbers represent the different MAC addresses per scan and not the physical APs. In the Figure, the ratio of the University owned APs of the TUnet network (orange bars) and all visible signals (blue bars) is shown. On average, 19 stationary AP signals from the University network per scan could be measured in the library and outside. At checkpoints 1 to 6 the difference between the signals from the TUnet and other signals is the largest. These points are located outdoors and many other external signals are received. When considering the frequency distribution, checkpoints 5 and 6 stand out, where an above-average number of signals per scan is observed. These two checkpoints are located directly in front of the library entrance, which is why many signals of the TUnet from the adjacent office building can also be received here. The low number of signals at checkpoint 19 results from the fact that this point is located at a corner of the room on the ground floor. Checkpoints 20 to 25—with the exception of point 23—also show a lower number of signals per scan. These checkpoints are located in the staircase, where there are no APs. Especially at checkpoint 24, very few signals with an average of 4.7 signals per scan were received.

### 3.3. Test Measurement Procedures

Test measurements were carried out in three different modes, i.e., static, stop-and-go and kinematic. In the case of static measurements, individual signal strengths measurements were carried out in several user orientations at the checkpoints. The necessary orientation measurements were performed in the possible movement directions. For example, only two orientations were measured in the corridors and four orientations at nodes (where two corridors intersect). At least 50 single Wi-Fi scans with several different smartphone models were measured at each checkpoint.

**Figure 3:**
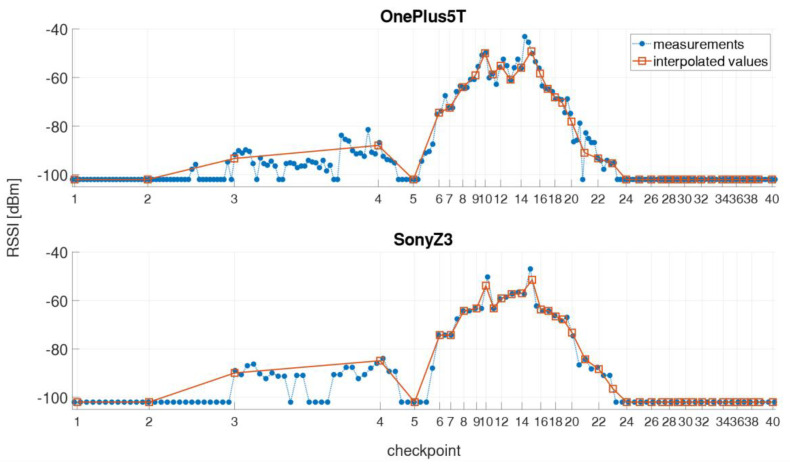
Linear interpolation of the kinematic measurements for the two smartphones (**top**) OnePlus 5T and (**bottom**) Sony Z3 which measured simultaneously.

In the kinematic measurement mode, the Wi-Fi RSSIs were continuously recorded along the defined trajectory while the user walked along with a usual walking speed of 1 ms^−1^ back and forth. The obvious advantage of this mode is that the time required for the off-line training phase is much shorter than for the static measurements, in which a measurement cycle took approximately 40 min compared to 4 min only in kinematic mode. However, this measurement procedure does not exactly carry out a Wi-Fi scan on every checkpoint due to time taken for a single Wi-Fi scan. Thus, the result significantly depends on the scan duration [1,4]. For the creation of the radio maps (see Section 4.2), however, the signal strengths on the checkpoints must be known, which is why the RSSI values of a measurement run have to be interpolated in time. Therefore, a timestamp was set on each checkpoint while passing by. The linearly interpolated RSSI values on the checkpoints can then be saved in the fingerprint database. Figure 3 illustrates a kinematic measurement process and the linear interpolation of signal strengths. If the signal of an AP is not measured during the scan, then a RSSI value of −102 dBm was assigned for the missing value. This value was chosen as, since the lowest value measured in the test site was −101 dBm. As already mentioned, each smartphone takes a certain amount of time to perform a Wi-Fi scan (compare Table 4). In Figure 3, therefore, the two smartphones with the shortest and the longest scanning time are presented, whereby the two smartphones performed the measurement simultaneously. Although higher number of scans, i.e., 201 scans, can be performed during the same time interval with the OnePlus 5T smartphone due to the shorter scanning time than with the Sony Z3 (115 scans), there is a great similarity between the two signal series. The signal strengths are derived from the 5 GHz Wi-Fi signal of the AP DDEG-2 in the case shown.

**Figure 4:**
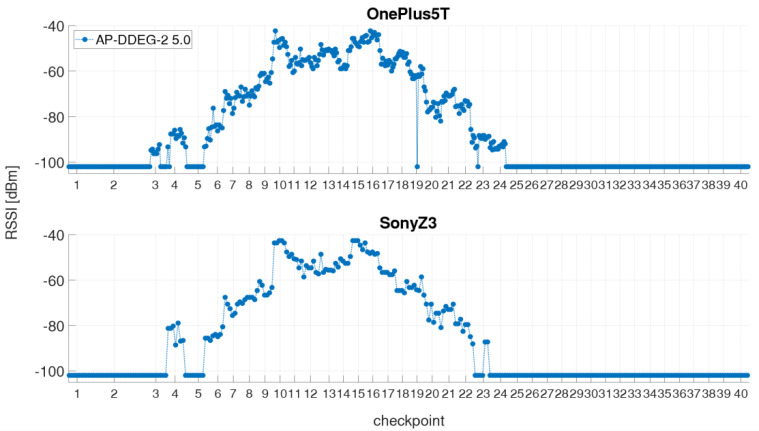
Examples of a stop-and-go measurement run for the two smartphones (**top**) OnePlus 5T and (**bottom**) Sony Z3 which measured simultaneously.

**Figure 5:**
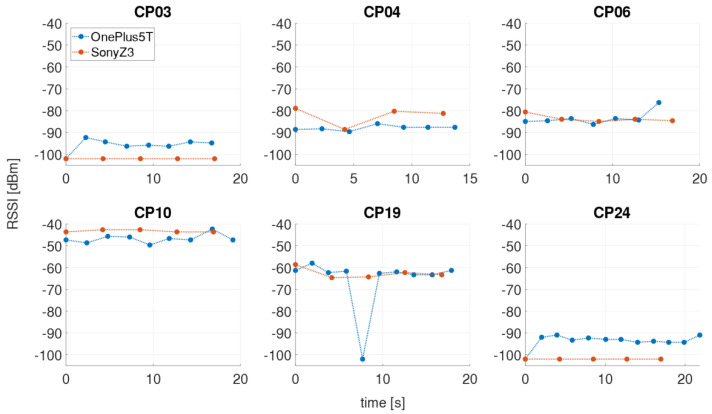
Zoomed views of the stopping phases for selected checkpoints for the two smartphones.

In the stop-and-go mode, measurements were carried out at each checkpoint for a certain period of time of approximately 20 s so that at least five Wi-Fi scans are available. Thus, in contrast to the kinematic mode no interpolation must be performed. As an example, Figure 4 shows a measurement run for the same two smartphones and AP as in Figure 3. Again a great similarity between the signal sequences of the two smartphones can be seen. For a more detailed analysis, the scans on selected checkpoints are magnified in Figure 5. Checkpoints 3 and 24 are the ones where the signal could be at first or at last received along the trajectory. These two checkpoints are also those with the lowest RSSIs. However, only with the OnePlus 5T smartphone the signal could be measured with RSSI values −94.9 dBm or −92.9 dBm, respectively. At checkpoint 10 the highest RSSI values can be measured with average signal strength of −44.7 dBm with the OnePlus 5T and −43.2 dBm with the Sony Z3. At checkpoint 19 it is noticeable that the signal could not be measured during a scan, although it was measured shortly before and after with approximately −62 dBm. Furthermore, it can be observed at all checkpoints that the signal strengths are not always stable, but differ slightly from scan to scan. In order to investigate how the RSSI values behave within this short period of time, the standard deviations were calculated. The largest signal fluctuations occurred at checkpoints 4 with ±4.3 dBm and 6 with ±3.3 dBm. Viewed over all measurements and checkpoints, the average standard deviation of the signal strengths during the stopping phase is only ±1.5 dBm.

## 4. Analyses of the Off-Line System Training Phase

Training measurements were carried out in front of the main building of the University, in the library and the Freihaus building along predefined trajectories with reference waypoints at decision points, such as trajectory crossings, and at irregular intervals depending on the local conditions. During the kinematic measurements, a time stamp was set at the waypoints when the user passed, in order to be able to interpolate the RSSI values at these points.

### 4.1. Measurement Mode Comparison

The fingerprint databases were created from the RSSI measurements of all smartphones either separately or combined. However, not every measurement is used separately, but the average RSSI values are collected for each checkpoint in a vector. In order to obtain a combined device-independent fingerprint database, a calibration with a multivariate linear regression as in [1] was carried out in the form of:

(1) $$y_{RSSI}=a_{S}\cdot x_{S}+b_{S}$$

where $x_{S}$ is the measured RSSI value from the smartphone S which should be calibrated, $y_{RSSI}$ the averaged reference vector calculated from all average RSSI values that are estimated with the linear regression model and $a_{S}$ and $b_{S}$ are the calibration coefficients. To obtain a gradient which is equal for each smartphone, $a_{S}=\mathrm{const}.$ is assumed in the linear regression model. The adjusted RSSIs using these calibration coefficients can then be used for a combined fingerprint database. Applied to these datasets, the variation range could be reduced from 27 to 16 dBm using the calibration. Overall, the average standard deviation of all measurements could be reduced from 4.2 to 3.0 dBm (for further details see [1]). In order to compare these measurement modes and the databases, the differences between the mean RSSI values were calculated for each checkpoint and AP.

**Figure 6:**
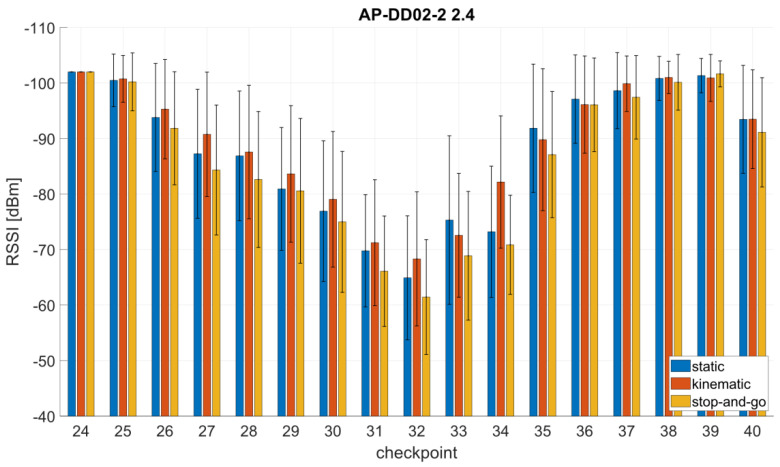
Mean RSSI and their standard deviations for comparison of the databases of the three measurement modes static, kinematic and stop-and-go.

Figure 6 shows the mean signal strengths and their standard deviations of one AP where the largest differences between the database values were found in the library for the three databases from the static, stop-and-go and kinematic off-line training measurements. Thereby the 2.4 GHz signal from the AP DD02-2 showed the largest difference at checkpoint 34. The average difference for this AP resulted in 0.8 dBm between the databases derived from static and kinematic training measurements, 1.0 dBm between the static and stop-and-go database and 1.5 dBm between the kinematic and stop-and-go database. The largest difference between the stop-and-go and the kinematic database reached 11.3 dBm. For further comparison, the correlation coefficients between pairs of the same APs were calculated. For this purpose, on the one hand, the database from the averaged RSSI was used, and on the other hand, their variances. The results for the mean correlation coefficients and differences between the databases are presented in Table 5. With regard to the RSSI values, the databases show nearly no differences and are highly correlated. The average difference between pairs of the same AP is also very low. In the case of the variances, the correlation with the kinematic measurements is somewhat weaker. This is probably due to the lower number of off-line measurements, i.e., 60 scans per checkpoint, in this measurement mode. All in all, however, the databases are very similar, which is why the databases are now combined for the subsequent creation of the radio maps and the analyses of the positioning results.

### 4.2. Radio Map Generation

In order to know the RSSIs and variances of the APs not only at the waypoints, but also in the whole test site, an area-wide interpolation is carried out for each AP for both the RSSI values and the variances. Different interpolation methods can be used for this purpose (see e.g., [45]). An interpolation by natural neighbors, also referred to as Voronoï interpolation, is used in this work [46,47,48]. The grid width of the interpolated radio maps is set to 1 m, which results in that positioning can be carried out within meter accuracy. In a multi-storey building, when creating the radio maps, it must be kept in mind that a separate radio map for each AP is created for each floor, always using only the RSSI measurements on those checkpoints that are located on the respective floor. The different radio maps of a floor can be combined into a three-dimensional array in the form of datacube (see Section 2.5), with the first two dimensions resulting from the extent of the floor and the third dimension from the number of APs.

The creation of an empirically determined radio map starts with the classification of reference points on the basis of a building map. Care should be taken to ensure that the reference points are well distributed throughout the building. In the off-line training phase, the signal strengths—derived from different APs—are then measured at each reference point. A fingerprint respective scan $s_{RP_{i},t}$, which was carried out at the reference point $RP_{i}$ at time t, is thus composed of the measured RSSI values $RSSI_{AP_{j}}$ of the N APs [1]:

(2) $$s_{RP_{i},t}=\left[\begin{array}{c} RSSI_{AP_{1}}\\ RSSI_{AP_{2}}\\ \begin{array}{c} \vdots \\ RSSI_{AP_{N}} \end{array} \end{array}\right]$$

The measured signal strengths are then assigned to the corresponding APs in the fingerprint database. To do this, however, it first has to be determined which APs are to be used for localization. If several scans are performed at a reference point, then the database consists of all the scans at each reference point. It can happen that the number of signals strengths received for each scan is different, because for example an AP temporarily does not broadcast a signal, or the signal is too weak to be sensed. This leads to problems in determining the position when RSSI values of different APs occur in the observed fingerprint and in the fingerprint in the radio map. Therefore, a constant RSSI value is used for the missing fingerprint in this work, which means that the signal of the AP was not measurable. As aforementioned here a constant minimum value of −102 dBm was used, since the lowest value ever sensed in the area was −101 dBm.

Not every scan is used separately for localization, but the RSSI averages of the measurements are collected in the vector given in Equation (2). A suitable reference value for the sensed RSSI values must be found for this purpose. If the measured values are assumed to be normally distributed and contain only random errors, then the mean value is an optimal reference value. The database’s fingerprint $f_{RP_{i}}$ consists of all the mean RSSI values sensed at that reference point in the form:

(3) $$f_{RP_{i}}=\left(f_{AP_{j}}\right)=\;\frac{1}{N}\overset{N}{\underset{t}{\sum }}RSSI_{t,AP_{j}}=\left[\begin{array}{c} {\bar{RSSI}}_{AP_{1}}\\ {\bar{RSSI}}_{AP_{2}}\\ \begin{array}{c} \vdots \\ {\bar{RSSI}}_{AP_{N}} \end{array} \end{array}\right]$$

Each reference point generally has a unique characteristic and therefore acts like a RSSI fingerprint (therefore also the name fingerprinting for the localization approach). Thereby each RSSI is measured with a certain precision at each reference point. The value for the precision of a measurement series is the variance or standard deviation. This information can also be used for fingerprinting by providing each fingerprint with its covariance matrix. The measurements are assumed to be uncorrelated, which means that the empirical covariance matrix $C_{ff}$ contains only the variances $s_{RSSI_{AP_{j}}}^{2}$ of the APs $AP_{j}$ in the diagonals as given in:

(4) $$C_{ff_{RP_{i}}}=\;\left[\begin{array}{c} s_{RSSI_{AP_{1}}}^{2}\\ 0\\ \vdots \\ 0 \end{array}\begin{array}{c} 0\\ s_{RSSI_{AP_{2}}}^{2}\\ \vdots \\ 0 \end{array}\begin{array}{c} \cdots \\ \cdots \\ \ddots \\ \cdots \; \end{array}\begin{array}{c} 0\\ 0\\ \vdots \\ s_{RSSI_{AP_{N}}}^{2} \end{array}\right]$$

The fingerprinting database thus contains a fingerprint $f_{RP_{i}}$ for each reference point and is thus a two-dimensional array with the reference points as columns and the APs as rows. To determine the position, either the database can be used directly, or a radio map for each AP can be created by means of surface interpolation of the RSSI values. For that purpose, however, the coordinates of the reference points must be known. The interpolated radio map of an AP then contains the mean RSSI values on the reference points as well as the interpolated RSSI values between them. Since an individual radio map is created for each AP, the radio maps can be combined into a three-dimensional array, with the first two dimensions resulting from the length and width of the building and the third dimension from the number of APs. For complex buildings, however, it is not necessary to create a single radio map array for the entire building, but it is also possible to create single arrays for certain areas (e.g., floors). The size of the radio maps depends on the grid size as well as the spatial conditions and influences the quality and duration of localization. The larger a radio map is, the longer it takes to determine the position of the user. The accuracy of the position determination depends mainly on the density of the APs and the quality of the radio maps. The quality of the radio maps deteriorates over time due to the fluctuations in the AP signals and changes in the environment, such as changes in the position of furniture or other objects. Therefore, it is important that new fingerprints are collected regularly in order to keep the radio maps up-to-date. In a previous study, the authors [45] have developed a continuous kinematic training of the fingerprint database where measurements along walked trajectories are used to update the database. An important finding in the investigation of the radio maps is that the database created either from static, stop-and-go and kinematic measurement modes show a great similarity in both RSSIs and variances. For future work, this also means that continuous kinematic system training can be carried out, which means that the training phase is much shorter.

**Figure 7:**
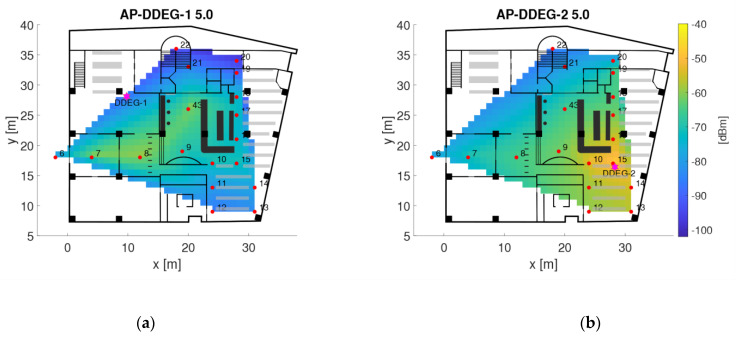
Radio maps for the two APs (**a**) AP-DDEG-1 and (**b**) AP-DDEG-2 on the ground floor of the library.

**Figure 8:**
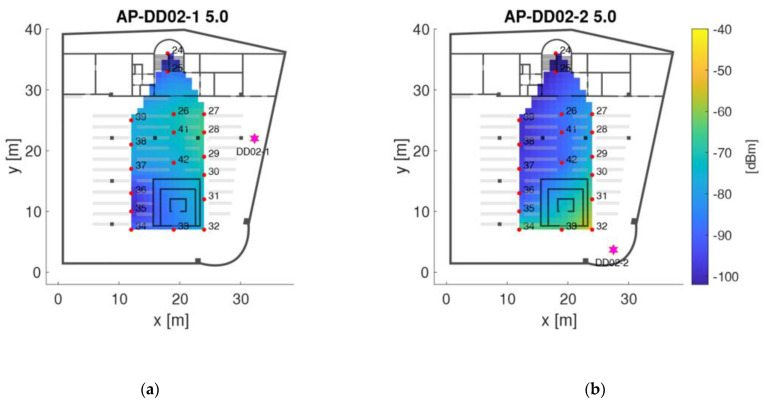

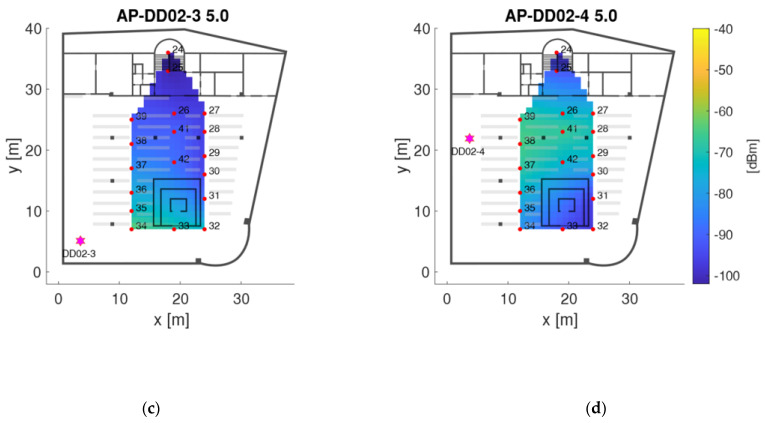
Radio maps for the four APs (**a**) AP_DD02-1, (**b**) AP-DD02-2, (**c**) AP-DD02-3 and (**d**) AP-DD02-4 on the second floor of the library.

As examples, Figure 7 and Figure 8 show the radio maps of the two APs on the ground floor and the four APs on the second floor, respectively. Thereby only the 5 GHz frequency band of the Wi-Fi signals is presented. In addition, own radio maps were created for the outdoor area. Since there is only one checkpoint on the first floor, no surface interpolation can be performed, which is why the radio map on this floor consist only of the fingerprint of checkpoint 23. The signals of 77 APs were used in total, which is why the radio map datacubes have the sizes presented in Table 6. From Figure 7 can be seen that the Wi-Fi signals can be received at all checkpoints on the ground floor. AP DDEG-1 can be sensed at checkpoints 7, 8 and 43 with the highest signal strength of approximately −60 dBm. Surprisingly, however, the RSSI values are quite low under this AP. This is most likely not the case, but is due to the fact that no measurements were carried out in this area around the AP. The signals of the AP DDEG-2 can be well received in the entire area. Since checkpoint 15 lies directly under this AP, the signal is received with a high average value of −48.6 dBm. The radio maps for the 5 GHz Wi-Fi signals of all four APs on the second floor are shown in Figure 8. All of them can be received at all checkpoints. However, the signals in the diagonally opposite corners are very weak, i.e., only −91 to −98 dBm. While cross-comparing Figure 7 and Figure 8 it is seen that the RSSI values are much lower on the second than on the ground floor. From Figure 8 can also be seen that the signal strengths were only interpolated between the checkpoints, which is why localization can only be performed in the inner area of the reading room between these bookshelves. If one would extrapolate the RSSI values outside this area, this would result in none realistic RSSI values as the signals could be determined either very strong or weak with high or low RSSI values, respectively. In order to have a radio map for the whole reading room, measurements not only on the selected checkpoints along the trajectory but also at additional points at room boundaries need to be carried out. The investigation in this paper, however, dealt with measurements in kinematic mode along the predefined trajectories to minimize the workload for system training. From the results presented in Section 4.3 one can see that the similar low signal strength values from all four APs led to lower positioning accuracies.

### 4.3. Visibility and Range of the Wi-Fi Signals

**Figure 9:**
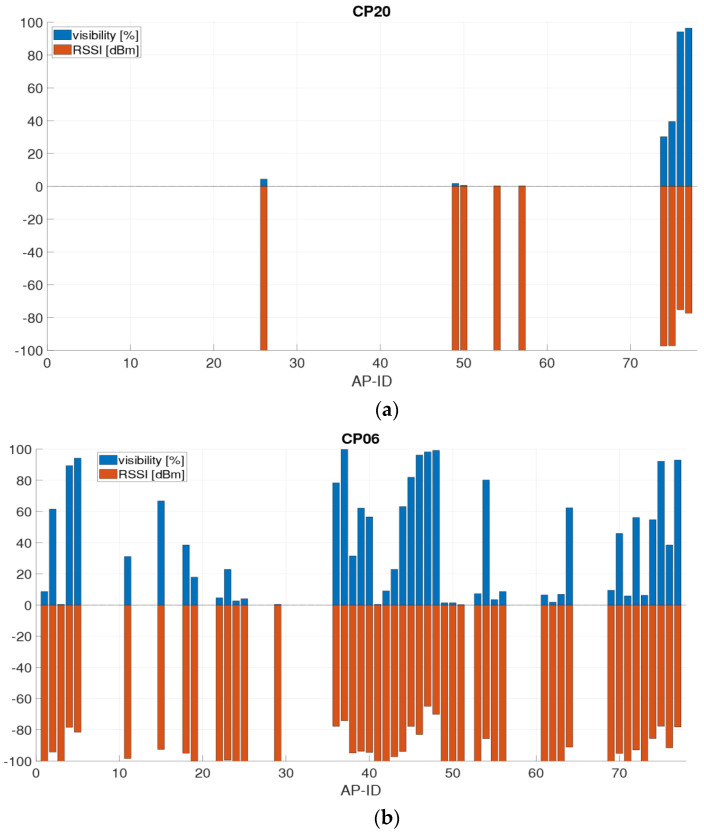
Checkpoints with (**a**) the lowest (CP20) and (**b**) highest (CP06) visibility.

As aforementioned, not all APs are detected at every scan. Figure 9a,b show, for example, the signal strengths and visibility of the APs at those checkpoints where to lowest and highest number of APs could be sensed. At checkpoint CP20 (Figure 9a), the signals of nine different APs were measured, of which only two were visible more than in 75% of the observations. CP20 is located quite remotely in a room corner on the ground floor of the staircase (compare Figure 1a). The two frequency bands of the AP DDEG-2 were most visible and were also received with higher signal strength. The 5 GHz signal was visible in 96% of the scans and had and average RSSI value of −77.4 dBm. Although the 2.4 GHz signal has a slightly lower visibility, i.e., 94%, it could be received with average RSSI values of −75.2 dBm. At checkpoint CP06 (Figure 9b), 47 different APs could be sensed at least once, of which many APs were visible in more than 75% of cases. CP06 is directly located at the library entrance. Here, the 5 GHz signals of the APs DCEG-3 and DC01-3 were the most visible with 99%. The 2.4 GHz signals of DCEG-3 have a visibility of 98% with the highest signal strength on average of −65.0 dBm. The 5 GHz signal, on the other hand, showed a −5.2 dBm lower mean signal strength of −70.2 dBm. Thus, a correlation between the visibility and the RSSI values is obvious. Therefore, the correlation coefficient of these two measured values was determined for each checkpoint; it resulted in 0.96. This means that the higher the signal strength of an AP, the more often this AP is also visible.

A further correlation exists between the two frequency bands 2.4 and 5 GHz that all APs provided. Across all checkpoints, the 2.4 GHz signals were on average 3.6 dBm higher than the 5 GHz signals. The average standard deviations resulted in ±4.5 dBm for the 2.4 GHz and ±3.5 dBm for the 5 GHz frequency band. In terms of signal range, the 2.4 GHz has a longer range from an AP compared to the 5 GHz band as it penetrates shielding materials with less loss and also has less free space path loss (FSPL), although the 5 GHz band has a 3.0 dBm higher transmitting power. The FSPL describes the reduction of the power density of an electromagnetic wave in free space, i.e., without interference from damping media, such as air or interference caused by reflections. As shown in [1], the attenuation thereby depends on the signal frequency and the signal weakens with increasing distance from the transmitter, also in terms of the signal-to-noise ratio. The FSPL in the unit dB is usually described on a logarithmic scale by means of the Friis transference equation [49]:

(5) $$FSPL\;\left[dB\right]=10\cdot \log_{10}{\left(\frac{4\cdot \pi \cdot d\cdot f}{c}\right)}^{2}$$

where d is the distance between the transmitter and receiver in [m], f is the frequency of the signals in [Hz], and c is the propagation speed in [ms^−1^]. Thus, the power decreases with the square of the distance to the transmitter. This applies for direct LoS signals. In practice, an empiric logarithmic distance model can be derived from Equation (5), because also with LoS signals, reflections and damping due to physical objects occur. Thereby, a Wi-Fi signal is already considerably attenuated within a few meters from an AP and the attenuation increases with the increasing frequency [1]. This mathematical relationship proves that the 5 GHz Wi-Fi signals have a shorter range that the 2.4 GHz signals.

**Figure 10:**
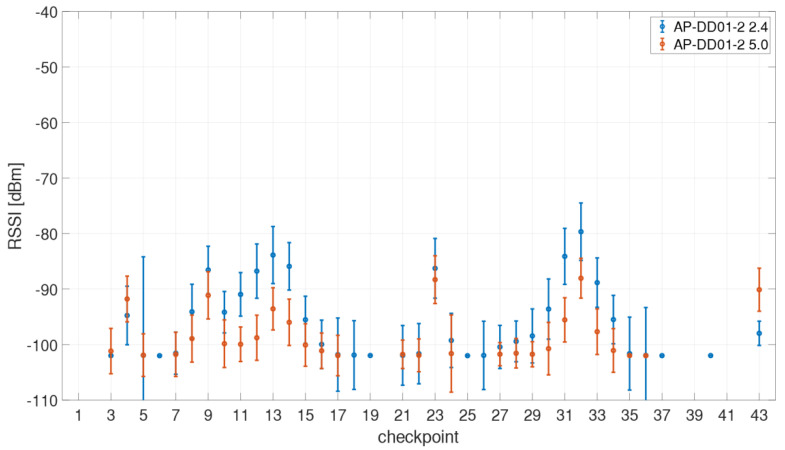
Sensed RSSIs of the AP DD01-2 from the first floor on all checkpoints for both the 2.4 and 5 GHz frequency bands.

Furthermore, the use of an AP from a different floor in the building was analyzed. For this purpose, one AP from the first floor of the library (between the two test areas) visible on most checkpoints was selected. The RSSI values of the AP DD01-2 for both the 2.4 and 5 GHz frequency bands are presented in Figure 10. They were received on 36 checkpoints with varying RSSI values. With an average of 79.7 dBm for the 2.4 GHz band and 88.0 dBm for the 5 GHz band the highest Wi-Fi signals were measured at checkpoint CP32 which is located directly above the AP on the second floor. This proves again that that there is a significant difference between the two frequency bands in terms of signal strength and range.

### 4.4. Kinematic System Training

Retscher and Hofer [50] introduced the checkpoint concept for Wi-Fi positioning. System training for fingerprinting is usually carried out in static mode on reference points distributed in a regular grid in the area of interest. The main disadvantage of this training procedure is therefore the required high workload. With trajectory checkpoints, the time needed for system training can be reduced by three quarters as shown in [50]. In the following steps of development, static training was replaced by kinematic measurements while walking along the trajectories [45]. Without stopping at reference points the user walks along predefined trajectories throughout the building. These kinematic measurements, however, pose much greater challenges than the usual static training measurements. As discussed in Section 3.3, the RSSI values on the checkpoints need to be interpolated from the whole RSSI time series. The reason for the required interpolation is that the RSSIs are continuously recorded and a single Wi-Fi scan takes some time. The scan duration depends heavily on the number of sensed APs and mostly on the hardware of the smartphone. In the following Section 5 this impact is discussed in further detail. Table 4 shows average scan durations for the employed smartphones in the test. One might think that the long times occurred only because of the fact that the smartphones used in the tests are quite old. The obtained different range of scan durations, however, is representative for a great variety of smartphones which are available on the market. They also cover a wide range of different hardware. As can be seen from Table 4, the scan durations varied between around 2.5 to over 4 s. The average number of sensed APs per scan was in the range of around 30 to 40 AP signals. The analysis of the system training measurements showed that there are sufficiently stable signals available everywhere on the campus to carry out a position determination using Wi-Fi fingerprinting. Retscher and Leb [45] could demonstrate that the achieved positioning accuracies for the kinematic system training are not much worse than with static measurements. The big advantage, however, is that the training phase is much shorter and continuous system training can also be carried out if needed.

## 5. Impact of Different Scan Durations on the Positioning Results

**Figure 11:**
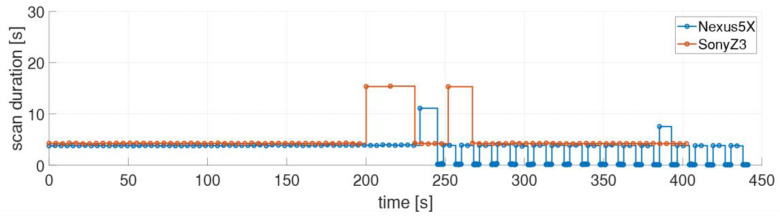
Irregular scan durations of the two smartphones Nexus 5X and Sony Z3.

Every smartphone needs a certain amount of time to perform a single Wi-Fi scan. These can be very different in length, as has been the case with the six different smartphones used (see Table 4). In Figure 3 the series of the two smartphones with the shortest and longest scan duration were presented in Section 3.3. A great similarity between the two time series can be observed although more scans along the trajectory can be performed with the OnePlus 5T smartphone than with the Sony Z3. As shown in this section, however, the scan duration has a significant influence for kinematic positioning in the on-line phase. If one looks at the whole collected dataset irregular scan durations were found for individual smartphones. They can deviate quite significantly from the mean scan durations presented in Table 4. Figure 11 shows such a case where two smartphones, i.e., the Nexus 5X and the Sony Z3, are compared. It can be seen that the Sony Z3 can have very long scanning times of even up to 15 s. The Nexus 5X, on the other hand, performs many scans with a measuring time of only a few milliseconds. These irregular scanning times are examined in more detail in the following.

**Figure 12:**
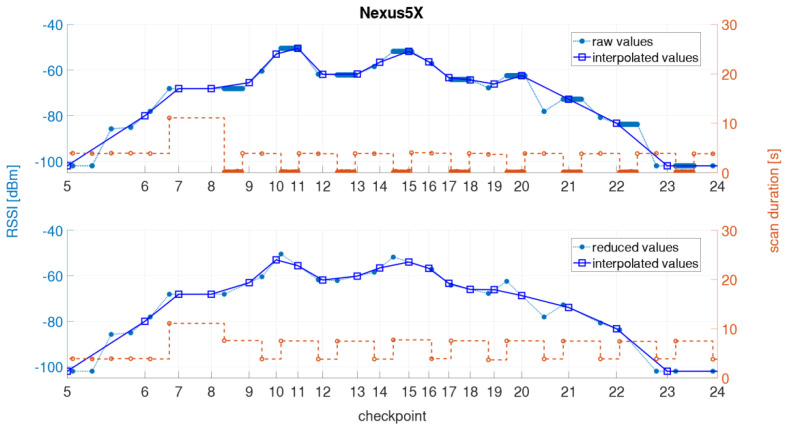
Kinematic measurements with the Nexus 5X smartphone with (**top**) raw and (**bottom**) interpolated RSSI values in dependence on the scan duration.

The short scan durations of the Nexus 5X are shown in Figure 12 together with the measured signal strengths. Again the results of the 5 GHz signal of the same AP as in Figure 3 are presented. As shown in Figure 12a, the irregular scanning periods start between the checkpoints 6 and 7. The pattern is always similar as first slightly longer scan duration occurs followed by a series of scans with a short scan duration, whereby the total duration of these scans corresponds to the average scan duration. After that, two scans occur with average scan duration and then a series of short scans starts again. A closer look indicated that during these short scan durations the RSSI values do not change which causes then problems in localization of the user. To reduce their effect, these scans were eliminated from the dataset. However, as a result a gap of one scan is present in the dataset as indicated in the Figure 12b. The reason for this effect, however, could not be clarified. It was only found with the Nexus 5X.

**Figure 13:**
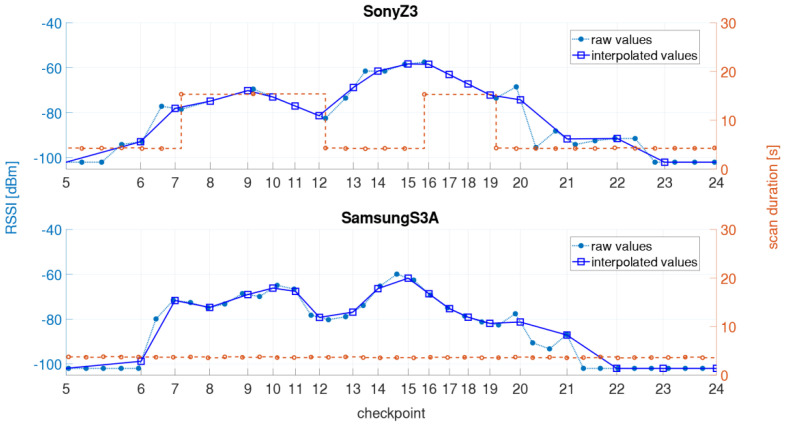
Raw and interpolated RSSI values of the two smartphones (**top**) Sony Z3 and (**bottom**) Samsung S3A in dependence on the scan duration.

Figure 13a shows the long scan durations of the Sony Z3 together with one of the Samsung S3, i.e., the S3A (Figure 13b). Both smartphones carried out the measurements at the same time. The Sony Z3 showed the longest scan durations near checkpoints 7, 9 and 16, which results that no Wi-Fi scan was performed along a distance 15 m while walking with an average speed was 1 ms^−1^. This leads to the fact that no scans are performed near checkpoints 8, 10, 11, 17 and 18. The interpolation can still provide similar values for the kinematic measurements as with the Samsung S3A phone. However, this does not apply in general. If, for example, no Wi-Fi scans were carried out between checkpoint 11 and 14, the interpolation would estimate too high RSSI values for the checkpoints in between. It was found that the smartphone tried to connect automatically to known Wi-Fi networks although it was first disconnected from the network. The connection function was disabled in order to have no influence from the signal strength changes while trying to connect on the positioning result. In the following, the maximum allowable scanning time for a meaningful interpolation was investigated. If there is long scan duration between two checkpoints then it has no influence on the interpolation. The maximum allowable scanning duration therefore depends on the spatial conditions, i.e., essentially on the distance between the checkpoints. If two checkpoints are located close to each other, it can be assumed that the signals show similar high values. If they are several meters apart, the RSSI values can vary significantly depending on the environment and the interpolation may no longer provide meaningful values. Since the fingerprint database in this work consists of many scans and these irregular scan durations only occurred in a few measurement runs, these scanning delays have no significant effect on the presented positioning results. If a long scan occurs in the on-line positioning phase, it is clear that no positioning can be carried out during this time, as no Wi-Fi RSSI values are available.

## 6. Localization in the On-Line Positioning Phase

For localization in the on-line positioning phase, RSSI measurements are carried out and matched to the fingerprint database. Most commonly either deterministic or probabilistic fingerprinting techniques based on pattern recognition are employed [2]. In this study, a probabilistic approach is applied as it provides, in general, higher positioning accuracies than deterministic methods in indoor positioning [51,52,53,54]. The main reason for this is that probabilistic fingerprinting accounts better for signal fluctuations. For the analyses of the achievable positioning accuracies, on-line measurements were carried out with all three measuring modes, i.e., in static, stop-and-go and kinematic mode. In the following, the operational principle of a simple and straightforward probabilistic fingerprinting approach is briefly reviewed and then the results for static and kinematic positioning modes are presented.

### 6.1. Probabilistic Fingerprinting Approach

A probabilistic fingerprinting approach was selected where the basic idea is to compute a conditional probability density function (PDF) of the unknown position (see e.g., [1,55]). Starting from Bayesian filtering, a dynamic system with measurement noise can be dealt with. The posterior PDF of the unknown positions can be derived using Bayes’ theorem (see e.g., [56,57]) and the measurements because of the fact that the fingerprints contain information about the signal characteristics. In this work, a probabilistic approach based on the derivation of the Mahalanobis distance is applied [58]. The Mahalanobis distance $d^{M}$ has the form [1]:

(6) $$d^{M}\left(f_{map}^{i},\;f_{obs}\right)={\left(f_{obs}\;-\;f_{map}^{i}\right)}^{T}C_{ff_{map,i}}^{-1}\left(f_{obs}\;-\;f_{map}^{i}\right)$$

where $f_{obs}$ is the current on-line RSSI measurement at the position $f_{map}^{i}$ in the fingerprint database (or radio map) and $C_{ff_{map,i}}$ its empirical covariance matrix.

Equation (6) means that the estimated reference point with the highest probability density is the point at which the Mahalanobis distance $d^{M}$ between the observed fingerprint and the fingerprint of the corresponding point in the fingerprinting database is the smallest. The advantage of using the Mahalanobis distance is the additional use of the covariance matrix $C_{ff_{map,i}}$, since the distance metric is adjusted using the covariance matrix. This is also a distance criterion for the fingerprint matching. As the inverse of the covariance matrix is the weight matrix, the weighted square sum of the RSSI differences (between off-line training and on-line positioning phase) is calculated for the Mahalanobis distance. Then the weights are inversely proportional to the variances of the corresponding fingerprints. In fact, the Euclidean vector distance most commonly used in the deterministic fingerprinting approach (see e.g., [58,59]) is a special case of the Mahalanobis distance, which occurs when the covariance matrix becomes the unit matrix. In a previous study of the authors of this contribution it was seen that the simple and straightforward calculation of the Mahalanobis distance while using kinematic system training yielded to comparable results as algorithms where a static or stop-and-go mode for localization is applied.

**Figure 14:**
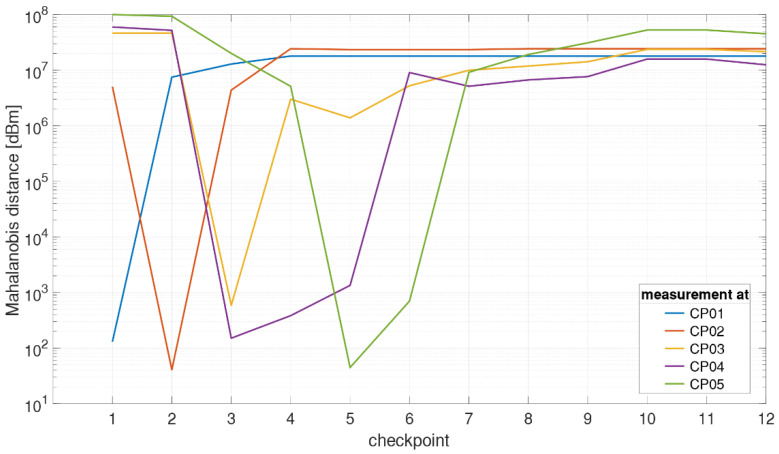
Positioning using the Mahalanobis distances at five different checkpoints (CP01 to CP05).

**Figure 15:**
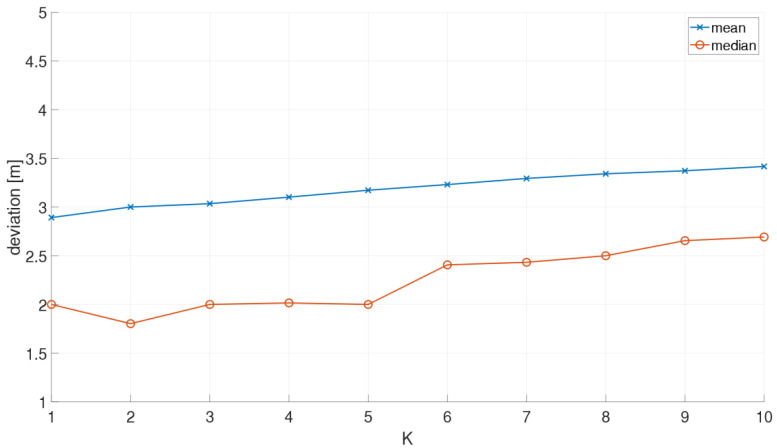
Mean and median deviations for static positioning in dependence of the K value for the nearest neighbor approach.

Its principle of operation is reviewed by giving a simple example. Figure 14 illustrates the position estimation for five on-line measurements. For each measurement at a checkpoint (CP), the Mahalanobis distance is estimated for each individual CP in the fingerprint database. The CP with the shortest distance is then the desired position. As shown in the Figure, the positions at CP01, CP02, CP04 and CP05 have been correctly determined. The on-line measurement at CP03, however, has its minimum at CP05, which means that the position in this on-line measurement has been indirectly determined. If one defines a so-called matching success rate (MSR) it would be zero for this checkpoint. This example shows the advantages of using the Mahalanobis distance for probabilistic fingerprinting. It is based on the knowledge of its covariance matrix. Standard deviations of each fingerprint must be known. In general, however, not all APs can be received anywhere in the measuring area, which can lead to problems with distance calculation if RSSI values are of different APs in the on-line and off-line fingerprint. As already mentioned, in the case a value of −102 dBm is used for the non-receivable AP. In the event that the signals of an AP could not be received at a single off-line measurement at a certain checkpoint—i.e., only values of −102 dBm are set at the corresponding location in the database—the variance is zero. However, the variance must not be zero, otherwise the determinant of the covariance matrix is also zero and thus the covariance matrix is singular and not invertible. However, the matrix must be inverted when calculating the Mahalanobis distance, see Equation (6). To avoid this problem, a variance of 0.0001 dBM is used in this case. If a signal from an AP can now be received in the on-line measurement at a point that could not be measured in the off-line phase, then the weighting becomes very large as the weighting is inversely proportional to the variance, which also increases the distance between the two fingerprints. As a result, then the likelihood that this point is the location one is looking for decreases. If the position is determined using interpolated radio maps, then the deviations of the calculated positions from the true position can be specified. The Mahalanobis distance between each point in the radio map and the on-line fingerprint is calculated first. Then, from the position in the radio map where the shortest distance was calculated (the nearest neighbor), the deviation from the true position is calculated using the Euclidean distance. Ideally, the Mahalanobis distances near the respective checkpoint are very short and grow with increasing distance. However, a set of K-smallest distances can also be selected to determine the position. This is referred to as K-nearest neighbor (KNN) approach. The searched position is then derived from the center of gravity of the K-nearest neighbors. Therefore, the static measurements were used to determine at which K the smallest deviations from the true position occur. In the library, the arithmetic mean of all deviations for K = 1 is 2.9 m and the median 2.0 m. As shown in Figure 15, the arithmetic mean and the median of all deviations in the measuring area increases the more neighbors for position determination are included. Therefore, only the nearest neighbor approach with K = 1 was applied in the further evaluation.

### 6.2. Static Positioning

For static localization, five RSSI scans were carried out in two different orientations—usually in the possible direction of movement along the trajectory—at each checkpoint. For the analysis, a matching success rate (MSR) was defined, i.e., how often the correct checkpoint in the on-line positioning phase was assigned. The achieved MSR was quite low of only 61% on average for all 43 checkpoints. Especially checkpoints at room borders and edges were determined with low MSRs. In addition, most of the incorrectly matched points were assigned to neighboring checkpoints. The test site was then divided into cells for cell-based localization (Figure 1 shows the cells with blue Roman numbers). If cell-based positioning is carried out then the MSRs can be significantly increased as indicated in Table 7. The two worst results were achieved in cells X and XI, which are either in the staircase or at the entrance to the second floor near the staircase. Here the adjacent cells are determined frequently. The two cells VI and VII located on the ground floor also showed low MSRs. In fact, this is caused by the usage of only two APs from the network of the University.

**Figure 16:**
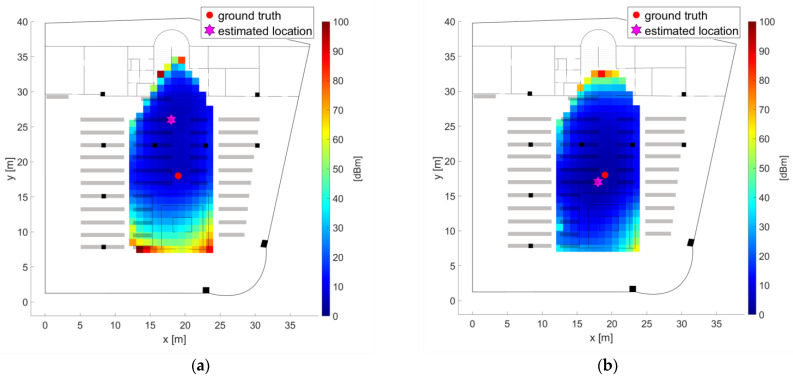
Two static positioning results on checkpoint 42 with the Samsung S3B with (**a**) worst and (**b**) overall best result.

For the following analyses, the positions were estimated on the basis of the interpolated radio maps, allowing the deviations of the calculated location to the ground truth to be determined. On the ground floor of the library, the average deviations from the ground truth resulted in 3.4 m and the median in 2.2 m. In particular, checkpoints CP11 to CP19 show above-average deviations of up to 5.0 m, as was also seen when looking at the MSR. This is caused again by the fact that there are only two APs on the ground floor and that there are no building structures that influence the Wi-Fi signals in such a way that the RSSI varies significantly on each checkpoint. On the second floor of the library, the positioning accuracies are better with mean deviations of 2.2 m and a median of 2.0 m. The largest deviations of 8.3 m resulted on CP42 in one measurement run with the Samsung S3B. Figure 16a shows the worst and Figure 16b the overall best result in localizing of this checkpoint. The estimated location resulted in a deviation of 1.4 m from the ground truth for the best solution. Here the difference of the Mahalanobis distance between the true location and the estimated position is approximately only 0.3 dBm. In the worst case, the Mahalanobis distances differ with values as large as 20 dBm. Further significant average deviations on this floor were seen at the two checkpoints CP27 and CP40. These two checkpoints have already achieved poor results when one looks at the MSR. CP27 and CP40 are located in the entrance area of the second floor near the staircase.

If one looks at the achieved results of the different smartphones, differences can be seen. Table 8 shows the statistical values for each of the six used smartphone in two orientations in the possible movement directions. As can be seen, the orientation of the user does not always play a major role for the resulting positioning accuracies. This is because of the fact that several orientations were measured for the off-line training measurements and thus the influence of the human body could be minimized. Table 8 also shows no major differences between the smartphones as they were calibrated with a linear regression model using the coefficients $a_{S}$ and $b_{S}$ obtained from Equation (1) (see Section 4.1).

### 6.3. Kinematic Positioning

**Figure 17:**
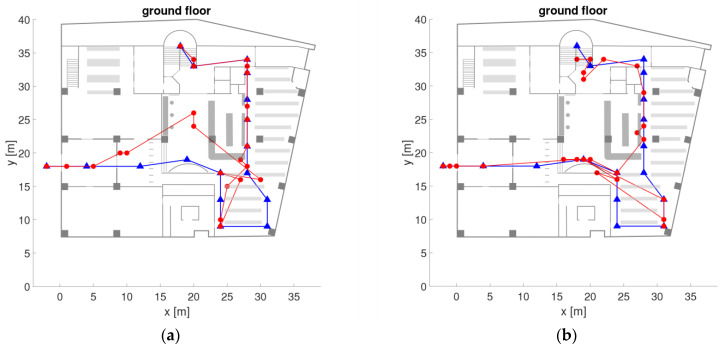
Trajectories of two measurement runs on the ground floor with estimated positions in red and ground truth in blue. (**a**) worst result; (**b**) good result.

**Figure 18:**
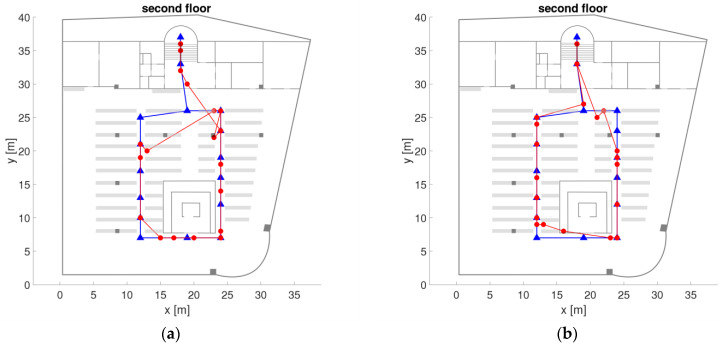
Trajectories of two measurement runs on the second floor with estimated positions in red and ground truth in blue. (**a**) worst result; (**b**) good result.

In the case of the on-line kinematic measurements, the user walked along the trajectories back and forth with usual step speed of approximately 1 ms^−1^. In the following, the results of 12 measurement runs are presented. Because each smartphone requires a certain amount of time to perform a single Wi-Fi scan, i.e., its scan duration, a fingerprint could not be taken exactly at every checkpoint as the user pressed only an event button when passing by at a certain checkpoint and did not stop at this point. In order to determine the deviations at the checkpoints, the RSSI values had to be interpolated linearly. In addition to the deviations of the estimated positions from the ground truth, the kinematic measurements also determine the positions along the whole trajectory for each single scan. This allows that the walked trajectories can be reconstructed. Figure 17 and Figure 18 therefore visualize the trajectories of two different measurement runs on the ground and second floor, respectively. Table 9 summarizes the deviations from the ground truth for the six different smartphones while walking back and forth. The deviations resulted in 2.7 m on average and a median of 1.4 m. As can be seen from Table 9, the largest mean deviation occurred with the Sony Z3 smartphone during the first measurement run with a value of 4.3 m. The reason for this large deviation is found in the long average scan duration of 4.1 s (compare with Table 4). Also the deviations of the Nexus 5X smartphone are larger which is also caused by the scan duration. As a result, the mesh points in the interpolation are lying further apart and their number is lower than as with short scan durations. Thus, the interpolation yield to a poorer approximation results in respective to the measured RSSI values. These results show that the time needed for a single Wi-Fi scan has a significant influence on the kinematic positioning results.

### 6.4. Cramér-Rao Lower Bound

To investigate the deviations from the ground truth in more depth the Cramér-Rao Lower Bound (CRLB) was calculated. The CRLB is defined as the inverse of the Fisher information matrix (FIM) F [60,61,62]:

(7) $$Cov\left(\theta \right)\geq F{\left(\theta \right)}^{-1}$$

The FIM F can be expressed as:

(8) $$F\left(\theta \right)=\left[\begin{array}{cc} F_{\mathrm{xx}} & F_{\mathrm{xy}}\\ F_{\mathrm{yx}} & F_{\mathrm{yy}} \end{array}\right]$$

with:

$$F_{\mathrm{xx}}\left(\theta \right)=\overset{m}{\underset{i=1}{\sum }}\rho \frac{{\left(x_{i}-x_{0}\right)}^{2}}{d_{i0}^{4}}$$

$$F_{\mathrm{xy}}\left(\theta \right)=F_{\mathrm{yx}}\left(\theta \right)=\overset{m}{\underset{i=1}{\sum }}\rho \frac{\left(x_{i}-x_{0}\right)\left(y_{i}-y_{0}\right)}{d_{i0}^{4}}$$

$$F_{\mathrm{yy}}\left(\theta \right)=\overset{m}{\underset{i=1}{\sum }}\rho \frac{{\left(y_{i}-y_{0}\right)}^{2}}{d_{i0}^{4}}$$

and the channel constant ρ:

(9) $$\rho ={\left(\frac{10\cdot n_{p}}{\sigma_{i}\cdot \ln10}\right)}^{2}$$

where $n_{p}$ is the path-loss exponent (typically between 2 and 4), $\sigma_{i}$ is the standard deviation of the RSSI of ${\mathrm{AP}}_{i}$, m is the number of APs and $d_{i0}$ represents the true distance between ${\mathrm{AP}}_{i}$ and the unknown mobile device, which is numbered as 0.

Finally, the lower bound on Root Mean Square Error (RMSE) can be computed by,

(10) $$\mathrm{RMSE}\geq \sqrt{\mathrm{trace}(F{\left(\theta \right)}^{-1})}$$

**Figure 19:**
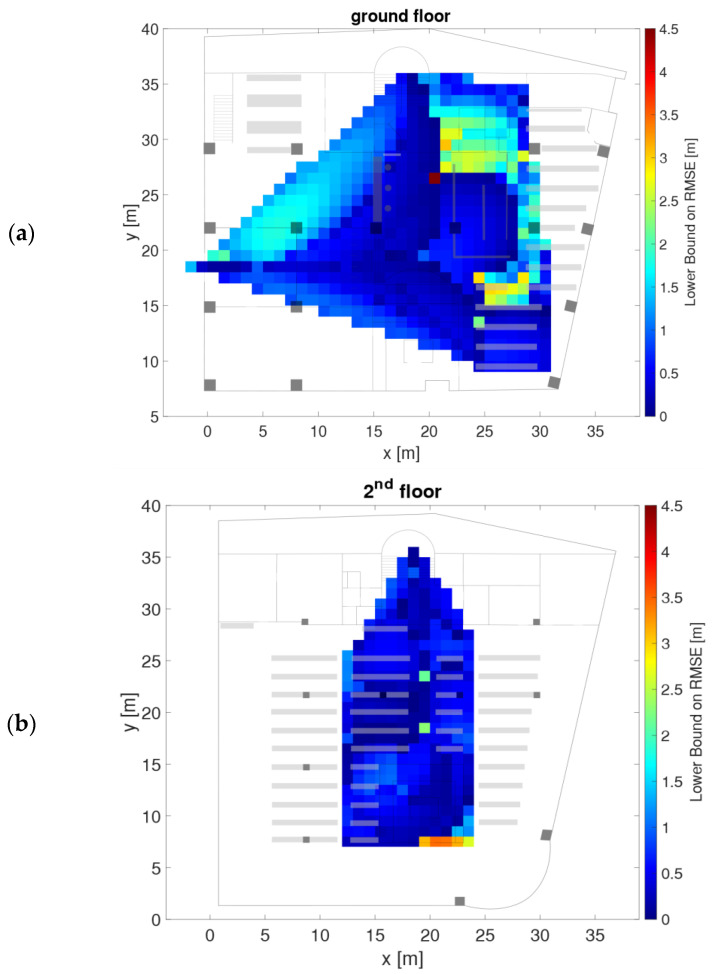
Visualization of the Cramér-Rao Lower Bound (CRLB) on the RMSE for (**a**) the ground and (**b**) the second floor.

Figure 19 shows a visualization of the resulting Cramér-Rao Lower Bound (CRLB) on the RMSE for the ground and the second floor in the library. Low CRLB values visualized in dark blue indicate higher positioning accuracies during the on-line phase, while higher values in red mean lower accuracy. Especially on the ground floor, one can see two areas where the CRLB is 2 to 3 m (green-yellow areas), while in the other parts of the area it has only values of 0.5 to 1 m.

### 6.5. Disscussion and Proposal for Performance Improvement

The results of the kinematic positioning tests indicate that the measured trajectories could be well reconstructed. Problems were seen only at the edges and in the corners on the ground floor and in the entrance area near the staircase on the second floor. If one compares the obtained results between the ground and second floor, however, differences in achievable positing accuracies can be seen. They are mainly caused by the existing building structures as the RSSI values do not vary significantly at the neighboring checkpoints on the ground floor. A significant difference on neighboring locations would facilitate a better matching success result. Although the second floor is a large reading room with many bookshelves, the resulting deviations on the checkpoints are smaller. The higher localization accuracies achieved could result from, on the one hand, the location of the bookshelves itself, which provide a significant variation of the RSSI values on the different checkpoints, and, on the other hand, due to the higher number of visible APs.

One major impact on the achievable positioning performance has not been considered so far. It relates to an optimization of the AP locations throughout the library. The current AP deployment enables only sufficient Wi-Fi communication services in most areas of the building. The APs are located in a rectangular shaped deployment at the same location on top of each other in every floor (apart from the ground floor). With AP rearrangement and additional deployment, it can be expected that higher positioning accuracies are achievable and a better service provided. Thus, future work will focus on this key point. It is proposed to deploy low-cost Raspberry Pi units serving as APs in addition. Retscher and Tatschl [63] have used Raspberry Pi units serving as APs and reference stations broadcasting as well as scanning and recording RSSI values at the same time. They introduced the Differential Wi-Fi (DWi-Fi) approach where reference stations as in Differential GNSS are deployed in the area of interest to derive correction parameters from the continuous sensed RSSI values of all involved APs and Raspberry Pi units (see also [1]). In Martínez-Gómez [64] Raspberry Pi units were employed as mobile devices. In our future research, it is planned to replace the smartphones by these devices in addition to the APs.

## 7. Path towards the Development of a Library Navigation and Information System

To assist students, staff and University visitors finding auditoriums and classrooms, offices and other rooms faster and easier, the positioning and navigation system can be combined with the in-house information system of TU Wien (TU Wien Information System & Services TISS) and with the e-learning platform TUWEL which is based on Moodle. Furthermore, additional application possibilities for location-related services are created. For instance, students could share their current location in order to be found faster by colleagues. A positioning system can also help to control and analyze people flows. These analyzes can later be a useful tool for, e.g., sustainable building development. In addition, short-term changes to the venue can be communicated more easily. The implementation of the presented positioning service at TU Wien can therefore lead to many new areas of application and thus contribute to an improvement of everyday life at the University. Especially for the library a navigation and information service is a very useful tool. To find a certain bookshelf it was seen in this study, however, that the integration with other technologies for positioning is required. Wi-Fi localization could be significantly improved if the new Round Trip Time (RTT) measurement protocol [65,66] is applied. In this case the double range between the transmitter, i.e., the AP, and the receiver, i.e., the smartphone, is derived by travel time measurement. Using RTT measurements ranges to the APs can be obtained with precisions on the decimeter level leading to higher localization accuracies than with common Wi-Fi fingerprinting [1,67]. The hardware of the APs, however, would need to be upgrade to be able to perform RTT measurements. Furthermore, currently not many smartphones on the market support these measurements. Another requirement would be to know the location of the AP precisely. If only the upgraded APs of the in-house Wi-Fi networks are used the location of the APs has to be surveyed once to obtain their 3D coordinates. The knowledge of the AP locations is not a requirement for location fingerprinting. Thus, a meaningful combination and integration of the RTT technology with fingerprinting is a promising solution. For further investigations the usage of low-cost Raspberry Pi units is foreseen. They should serve as APs and mobile devices enabling fingerprinting as well as RTT measurements.

Another improvement of localization performance in the kinematic positioning mode shall be achieved by the additional usage of the inertial sensors of the mobile devices. With smartphone accelerometers the distance travelled can be derived and with a gyroscope together with a magnetometer the direction of movement. Further developments are therefore focused on the integration of these sensors for continuous user localization.

Furthermore, other ways for the calibration of the RSSI recordings of different smartphone will be addressed in our future research. Apart from the calibration using a multivariate linear regression model the use of an in-motion calibration approach as in [68] shall be applied in order to cope with the inherent noise of Wi-Fi signals. Applying a window moving average filter to the raw RSSI recordings would lead to an improvement of the results.

A further task of investigation in this study is the integration with other technologies, such as Bluetooth LE beacons for areas with limited Wi-Fi coverage and serving as a backup solution as well as the usage of the RFID (Radio Frequency Identification) technology for book labeling and tracking. Thus, it is then possible to locate the correct book in the bookshelf itself and even detect if a book is taken out of the library without permission. RFID can be easily used for book location and tracking as books can be labeled with very cheap passive tags.

At TU Wien, however, it is a requirement that the navigation and information system should not cause high additional costs for, e.g., for installation of new hardware. Furthermore, a wide variety of mobile devices should be capable to use the service. These two requirements were the main reasons why so far only Wi-Fi fingerprinting was considered in the first stage of this study.

## 8. Concluding Remarks and Outlook

The investigations in this study have shown that Wi-Fi fingerprinting can be used to achieve positioning accuracies on the meter level in the library building of TU Wien and that the direction taken is useful for the development of navigation and information services. It is expected that the positioning accuracies in the library can be increased by installation of additional APs under consideration of their deployment to provide a better distribution and geometry for localization. Since the APs on the upper floors of the library are all arranged in a rectangle deployment, the question can also be asked whether a rearrangement can improve positioning accuracy. The optimization of the geometry of the AP locations is especially a crucial requirement if new technologies, such as Wi-Fi RTT measurements, shall be employed for increasing the positioning accuracies and service performance. Additional deployment of hardware is foreseen in the future by using low-cost Raspberry Pi units broadcasting and receiving Wi-Fi signals.

To overcome a major disadvantage of location fingerprinting concerning the required labor-intensive system training, new approaches, such as the usage of crowdsourced RSSI data (see e.g., [69,70,71,72]) from all service users, will be employed. For crowdsourcing, users can provide their scanned Wi-Fi RSSI values to build-up and continuously update the fingerprinting database. As the comparison of the different measurement modes—static, stop-and-go and kinematic—in the off-line training phase has shown, the database creation in kinematic mode and the achievable positioning accuracies differ not much from the other two measurement modes. This means, that continuous system training can be carried out, which reduces the time required.

## Figures and Tables

**Table 1:** Positioning technologies comparison.

Technology		Advantages	Disadvantages	Costs
optical	infrared	low power consumption	do not penetrate walls; susceptible to interference; low range	low
	visible light	low power consumption; high accuracy	do not penetrate walls; susceptible to interference	low
acoustic-based	audible	no costs	disruptive in everyday life	none
	ultra-sonic	high accuracy	susceptible to interference	medium
radio frequency	Wi-Fi	use of available infrastructure; accuracy on the m-level	susceptible to signal fluctuation; high power consumption	medium
	Bluetooth	low power consumption	susceptible to signal fluctuation and interference	low
	RFID	cheap passive tags can be mounted everywhere	low range and accuracy	medium
	UWB	multipath resistant; low energy consumption; high accuracy	expensive hardware	high
magnetic	natural	no costs	susceptible to interference	low
	artificial	low fluctuations	susceptible to interference	high
smartphonesensors	GNSS	freely available	not useable in buildings	none
	inertial	work independently	high sensor drift	none
	camera	works independently; visual information	computationally high costs	none

**Table 2:** Characteristics of positioning methods.

	Measurement Principle	Advantages	Disadvantages	Positioning Accuracy
CoO	cell-based	simple algorithm	relative positioning to location of transmitter	cell size dependent
Lateration	ToA, TDoA, RTT, RSSI-based	no off-line training phase	susceptible to multipath; LoS requirement	dm–m
Angulation	AoA	no off-line training phase	susceptible to multipath; LoS requirement; antenna array needed	dm–m
Fingerprinting	RSSI	no multipath influence; no LoS requirement	off-line training phase	m
Scene Analysis	-	no multipath influence	off-line training phase; high data transfer rates required; computationally intensive	dm–m
Dead Reckoning	-	only smartphone sensors used	relative positioning; large drift rates	m

**Table 3:** Characteristics and properties of range-based localization techniques for lateration.

	ToA	RTT	TDoA	RSSI-Based
signal propagation	does not matter	does not matter	does not matter	matters
LoS	required	required	required	not required
multipath	matters	matters	matters	partially matters
time synchronisation	transmitter and receiver	transmitter and responder	transmitter and receiver	not required
positioning accuracy	dm–m	dm	dm–m	m

**Table 4:** Average scan durations and sensed AP signals per scans for the six available smartphones.

	Scan Duration [s]	Average AP Signals per Scan
Nexus 5X	3.8	40.8
OnePlus 5T	2.4	42.6
Samsung S3A	3.5	33.7
Samsung S3B	3.5	27.5
Samsung S7	2.5	39.5
Sony Z3	4.1	39.3

**Table 5:** Mean correlation coefficients and variances for the comparison of the different fingerprint databases.

	RSSI		Variances	
	$\bar{r}$	$\bar{d}$ [dBm]	$\bar{r}$	$\bar{d}$ [dBm]
static—kinematic	0.96	0.3	0.93	3.9
static—stop-and-go	0.99	0.3	0.97	2.8
kinematic—stop-and-go	0.96	0.4	0.94	3.4

**Table 6:** Sizes of the radio map datacube arrays.

Outdoor	32×172×77
Ground floor	34×28×77
1st floor	1×1×77
2nd floor	13×30×77

**Table 7:** Matching success rates (MSR) in the cells in the library (for the location of the cells see Figure 1).

Cell	Checkpoints	Location	MSR
I	1, 2	outdoor 1	100.0%
II	3, 4	outdoor 2	100.0%
III	5, 6	entrance area (outdoor)	100.0%
IV	7, 8	entrance area (indoor)	66.7%
V	9, 43	ground floor lobby	87.5%
VI	10–15	ground floor area 1	56.9%
VII	16–19	ground floor area 2	54.2%
VIII	20–22	ground floor staircase	77.8%
IX	23	first floor staircase	100.0%
X	24, 25	second floor staircase	50.0%
XI	26, 27, 39, 40	second floor area 1	39.6%
XII	28, 29	second floor area 2	91.7%
XIII	30, 31	second floor area 3	66.7%
XIV	32–34	second floor area 4	100.0%
XV	35, 36	second floor area 5	70.8%
XVI	37, 38	second floor area 6	87.5%
XVII	41, 42	second floor area 7	66.7%

**Table 8:** Deviations in [m] from the ground truth in dependence of the smartphone for the static measurements.

Smartphone	Orientation	Mean	Median	Standard Deviation
**Nexus 5X**	1	4.2	3.0	4.0
	2	3.1	2.2	2.6
**OnePlus 5T**	1	3.5	3.0	3.5
	2	3.1	2.0	4.1
**Samsung S3A**	1	1.8	1.0	2.3
	2	2.1	1.0	3.6
**Samsung S3B**	1	2.6	1.0	3.8
	2	2.9	1.0	4.4
**Samsung S7**	1	3.3	2.2	3.4
	2	2.8	1.4	3.1
**Sony Z3**	1	3.1	2.0	6.4
	2	2.1	1.0	4.2

**Table 9:** Deviations in [m] from the ground truth in dependence of the smartphone for the kinematic measurement runs.

Smartphone	CP Start-End	Mean	Median	Standard Deviation
Nexus 5X	1-40	3.0	2.1	3.0
	40-1	2.9	2.2	2.7
OnePlus 5T	1-40	2.1	1.0	2.4
	40-1	1.9	1.0	2.7
Samsung S3A	1-40	1.6	1.0	2.3
	40-1	3.3	2.0	3.7
Samsung S3B	1-40	2.2	1.0	4.5
	40-1	2.9	1.0	5.0
Samsung S7	1-40	2.7	1.5	3.1
	40-1	2.0	1.0	2.4
Sony Z3	1-40	4.3	3.6	5.2
	40-1	3.9	3.0	3.9

## Data Availability

The data presented in this study are available on request from the corresponding author.

## References

[1] Retscher G. (2020). Fundamental concepts and evolution of Wi-Fi user localization: An overview based on different case studies. Sensors.

[2] Chen R., Pei L., Liu J., Leppäkoski H. (2012). WLAN and Bluetooth positioning in smart phones. Ubiquitous Positioning and Mobile Location-Based Services in Smart Phones.

[3] Liu H., Darabi H., Banerjee P., Liu J. (2007). Survey of wireless indoor positioning techniques and systems. IEEE Trans. Syst. Man Cybern. Part C Appl. Rev..

[4] Ali W.H., Kareem A.A., Jasim M. (2019). Survey on wireless indoor positioning systems. Cihan Univ. Erbil Sci. J..

[5] Youssef M., Agrawala A., Shankar A.U. WLAN location determination via clustering and probability distributions. Proceedings of the First IEEE International Conference on Pervasive Computing and Communications (PerCom 2003).

[6] Sakpere W., Adeyeye-Oshin M., Mlitwa N.B. (2017). A state-of-the-art survey of indoor positioning and navigation systems and technologies. S. Afr. Comput. J..

[7] Want R., Hopper A., Falcao V., Gibbons J. (1992). The Active Badge location system. ACM Trans. Inf. Syst..

[8] Yi K.Y., Kim D.Y., Yi K.M. (2015). Development of a Localization System based on VLC technique for an indoor environment. J. Electr. Eng. Technol..

[9] Akiyama T., Sugimoto M., Hashizume H. Time-of-Arrival-based smartphone localization using Visible Light Communication. Proceedings of the International Conference on Indoor Positioning and Indoor Navigation (IPIN 2017).

[10] Jung S.-Y., Hann S., Park C.-S. (2011). TDOA-based optical wireless indoor localization using LED ceiling lamps. IEEE Trans. Consum. Electr..

[11] Brena R.F., García-Vázquez J.P., Galván-Tejada C.E., Muñoz-Rodriguez D., Vargas-Rosales C., Fangmeyer J. (2017). Evolution of indoor positioning technologies: A survey. J. Sens..

[12] Moutinho J., Araújo R., Freitas D. (2016). Indoor localization with audible sound—Towards practical implementation. Pervasive Mobile Comput..

[13] Li J., Han G., Zhu C., Sun G. (2016). An indoor ultrasonic positioning system based on TOA for Internet of Things. Mobile Inf. Syst..

[14] Nakashima Y., Kaneto R., Babaguchi N. (2011). Indoor positioning system using digital audio watermarking. IEICE Trans. Inf. Syst..

[15] Lopes S.I., Vieira J.M.N., Reis J., Albuquerque D.F., Carvalho N.B. (2015). Accurate smartphone indoor positioning using a WSN infrastructure and non-invasive audio for TDoA estimation. Pervasive Mobile Comput..

[16] Woodman O., Harle R. Concurrent scheduling in the Active Bat location system. Proceedings of the 8th IEEE International Conference on Pervasive Computing and Communications Workshops (PERCOM Workshops 2010).

[17] Minami M., Fukuju Y., Hirasawa K., Yokoyama S., Mizumachi M., Morikawa H., Aoyama T. DOLPHIN: A practical approach for implementing a fully distributed indoor ultrasonic positioning system. Proceedings of the Ubiquitous Computing UbiComp 2004.

[18] Priyantha N.B., Chakraborty A., Balakrishnan H. The Cricket location-support system. Proceedings of the 6th annual international conference on Mobile computing and networking (MobiCom 2000).

[19] Batistic L., Tomic M. Overview of indoor positioning system technologies. Proceedings of the 41st International Convention on Information and Communication Technology, Electronics and Microelectronics (MIPRO 2018).

[20] Cominelli M., Patras P., Gringoli F. Dead on arrival: An empirical study of the Bluetooth 5.1 positioning system. Proceedings of the 13th International Workshop on Wireless Network Testbeds, Experimental Evaluation & Characterization (WiNTECH '19).

[21] Faragher R., Harle R. (2015). Location fingerprinting with Bluetooth Low Energy Beacons. IEEE J. Sel. Areas Commun..

[22] Zhao X., Xiao Z., Markham A., Trigoni N., Ren Y. Does BTLE measure up against WiFi? A comparison of indoor location performance. Proceedings of the 20th European Wireless Conference.

[23] Mainetti L., Patrono L., Sergi I. A survey on indoor positioning systems. Proceedings of the 22nd International Conference on Software, Telecommunications and Computer Networks (SoftCOM 2014).

[24] Seco F., Jimenez A.R. (2018). Smartphone-based cooperative indoor localization with RFID technology. Sensors.

[25] Großwindhager B., Stocker M., Rath M., Boano C.A., Römer K. SnapLoc: An ultra-fast UWB-based indoor localization system for an unlimited number of tags. Proceedings of the 18th International Conference on Information Processing in Sensor Networks (IPSN '19).

[26] Sadowski S., Spachos P. (2018). RSSI-based indoor localization with the Internet of Things. IEEE Access.

[27] Yeh S.-C., Hsu W.-H., Lin W.-Y., Wu Y.-F. (2020). Study on an indoor positioning system using Earth’s magnetic field. IEEE Trans. Instrum. Meas..

[28] Sheinker A., Ginzburg B., Salomonski N., Frumkis L., Kaplan B.-Z., Moldwin M.B. (2016). A method for indoor navigation based on magnetic beacons using smartphones and tablets. Measurement.

[29] Retscher G., Grafarend E.W. (2016). Indoor navigation. Encyclopedia of Geodesy, Earth Sciences Series.

[30] Gerstweiler G., Vonach E., Kaufmann H. (2016). HyMoTrack: A mobile AR navigation system for complex indoor environments. Sensors.

[31] Liu J., Chen R., Pei L., Guinness R., Kuusniemi H. (2012). A hybrid smartphone indoor positioning solution for mobile LBS. Sensors.

[32] Ettlinger A., Neuner H.-B., Burgess T. (2018). Development of a Kalman Filter in the Gauss-Helmert model for reliability analysis in orientation determination with smartphone sensors. Sensors.

[33] Retscher G. (2007). Augmentation of indoor positioning systems with a barometric pressure sensor for direct altitude determination in a multi-storey building. Cartogr. Geogr. Inf. Sci..

[34] Ye H., Gu T., Tao X., Lu J. (2016). Scalable floor localization using barometer on smartphone. Wirel. Commun. Mob. Comput..

[35] Werner M., Kessel M., Marouane C. Indoor positioning using smartphone camera. Proceedings of the International Conference on Indoor Positioning and Indoor Navigation (IPIN 2011).

[36] Mautz R. (2012). Indoor Positioning Technologies. Habilitation Thesis.

[37] Scaramuzza D., Fraundorfer F. (2011). Visual odometry [Tutorial]. IEEE Robot. Autom. Mag..

[38] Ramezani M., Acharya D., Gu F., Khoshelham K. Indoor positioning by visual-inertial odometry. Proceedings of the ISPRS Annals of Photogrammetry, Remote Sensing and Spatial Information Sciences.

[39] Aqel M., Marhaban M.H., Saripan M.I., Ismail N. (2016). Review of visual odometry: Types, approaches, challenges, and applications. SpringerPlus.

[40] Sahdev R., Chen B.X., Tsotsos J.K. Indoor localization in dynamic human environments using visual odometry and global pose refinement. Proceedings of the 15th Conference on Computer and Robot Vision (CRV 2018).

[41] Retscher G., Tatschl T. (2017). Indoor positioning with differential Wi-Fi lateration. J. Appl. Geod..

[42] Chen X., Kong J., Guo Y., Chen X. An empirical study of indoor localization algorithms with densely deployed APs. Proceedings of the Global Communications Conference GLOBECOM.

[43] Schnabel P. Elektronik-Kompendium. https://www.elektronik-kompendium.de/.

[44] RTR-GmbH RTR—Frequenzbereiche. https://www.rtr.at/de/tk/FRQ_spectrum.

[45] Retscher G., Leb A. Influence of the RSSI scan duration of smartphones in kinematic Wi-Fi fingerprinting (paper 9743). Proceedings of the FIG Working Week.

[46] LeDoux H., Gold C., Fisher P.F. (2005). An Effcient natural neighbour interpolation algorithm for geoscientific modelling. Developments in Spatial Data Handling.

[47] Lee M., Han D. (2012). Voronoi tessellation based interpolation method for Wi-Fi radio map construction. IEEE Commun. Lett..

[48] Üreten S., Yongaçoğlu A., Petriu E. A Comparison of interference cartography generation techniques in cognitive radio networks, In Proceedings of the 2012 IEEE International Conference on Communications (ICC), Ottawa, ON, Canada, 10–15 June 2012.

[49] Kaemarungsi K., Krishnamurthy P. Properties of indoor received signal strength for WLAN location fingerprinting. Proceedings of the Mobile and Ubiquitous Systems: Networking and Services MOBIQUITOUS 2004.

[50] Retscher G., Hofer H. (2017). Wi-Fi location fingerprinting using an intelligent checkpoint sequence. J. Appl. Geod..

[51] Bahl P., Padmanabhan V. RADAR: An in-building RF-based user location and tracking system. Proceedings of the 19th Annual Joint Conference of the IEEE Computer and Communications Societies INFOCOM 2000.

[52] Honkavirta V., Perälä T., Ali-Löytty S., Piché R. A comparative survey of WLAN location fingerprinting methods. Proceedings of the IEEE 6thWorkshop on Positioning Navigation and Communication WPNC’09.

[53] Dawes B., Chin K.-W. (2011). A comparison of deterministic and probabilistic methods for indoor localization. J. Syst. Softw..

[54] King T., Kopf S., Haenselmann T., Lubberger C., Effelsberger W. COMPASS: A probabilistic indoor positioning system based on 802.11 and digital compasses. Proceedings of the First ACM Workshop on Wireless Network Testbeds, Experimental Evaluation and Characterization (WiNTECH).

[55] Roos T., Myllymaki P., Tirri H. (2002). A statistical modeling approach to location estimation. IEEE Trans. Mob. Comput..

[56] Gordon N., Salmond D., Smith A. (1993). Novel approach to nonlinear/non-Gaussian Bayesian state estimation. IEE Proc. F.

[57] Koch K.-R. (2000). Einführung in Die Bayes-Statistik.

[58] Yeung W.M., Zhou J., Ng J.K.-Y. (2007). Enhanced fingerprint-based location estimation system in wireless LAN environment. Lecture Notes in Computer Science, Proceedings of the Emerging Directions in Embedded and Ubiquitous Computing Conference EUC 2007, Taipei, Taiwan, 17–20 December 2007.

[59] Moghtadaiee V., Dempster A.G. Vector distance measure comparison in indoor location fingerprinting. Proceedings of the International Global Navigation Satellite Systems IGNSS 2015 Conference.

[60] Patwari N., Ash J.N., Kyperountas S., Hero A.O., Moses R.L., Correal N.S. (2005). Locating the nodes: Cooperative localization in wireless sensor networks. IEEE Signal Process. Mag..

[61] Laitinen E., Lohan E. Access Point topology evaluation and optimization based on Cramér-Rao Lower Bound for WLAN indoor positioning. Proceedings of the 2016 International Conference on Localization and GNSS (ICL-GNSS).

[62] Li Q., Li W., Sun W., Li J., Liu Z. Cramér-Rao Bound analysis of Wi-Fi indoor localization using fingerprint and assistant nodes. Proceedings of the 2017 IEEE 86th Vehicular Technology Conference (VTC-Fall).

[63] Retscher G., Tatschl T. Differential Wi-Fi—A novel approach for Wi-Fi positioning ising lateration. Proceedings of the FIG Working Week.

[64] Martínez-Gómez J., Martínez M., Castillo-Cara M., Brea V.M., Orozco-Barbosa L., García I. (2016). Spatial statistical analysis for the design of indoor particle-filter-based localization mechanisms. Int. J. Distributed Sens. Netw..

[65] Van Diggelen F., Want R., Wang W. (2018). How to achieve 1-m accuracy in Android.

[66] Ibrahim M., Liu H., Jawahar M., Nguyen V., Gruteser M., Howard R., Bai F. Verification: Accuracy evaluation of WiFi fine time measurements on an open platform. Proceedings of the 24th Annual International Conference on Mobile Computing and Networking MobiCom '18.

[67] Guo G., Chen R., Ye F., Peng X., Liu Z., Pan Y. (2019). Indoor smartphone localization: A hybrid WiFi RTT-RSS ranging approach. IEEE Access.

[68] del Horno M.M., García-Varea I., Orozco Barbosa L. (2019). Calibration of Wi-Fi-based indoor tracking systems for Android-based smartphones. Remote Sens..

[69] Kim W., Yang S., Gerla M., Lee E.-K. (2016). Crowdsource based indoor localization by uncalibrated heterogeneous Wi-Fi devices. Mob. Inf. Syst..

[70] Rai A., Chintalapudi K.K., Padmanabhan V.N., Sen R. Zee: Zero-effort crowdsourcing for indoor localization. Proceedings of the 18th Annual International Conference on Mobile Computing and Networking Mobicom’12.

[71] Wu C., Yang Z., Liu Y. (2015). Smartphones based crowdsourcing for indoor localization. IEEE Trans. Mob. Comput..

[72] Zhou B., Li Q., Mao Q., Tu W., Zhang X., Chen L. (2015). ALIMC: Activity landmark-based indoor mapping via crowdsourcing. IEEE Trans. Intell. Transp. Syst..

